# An Evolutionary Model-Based Algorithm for Accurate Phylogenetic Breakpoint Mapping and Subtype Prediction in HIV-1

**DOI:** 10.1371/journal.pcbi.1000581

**Published:** 2009-11-26

**Authors:** Sergei L. Kosakovsky Pond, David Posada, Eric Stawiski, Colombe Chappey, Art F.Y. Poon, Gareth Hughes, Esther Fearnhill, Mike B. Gravenor, Andrew J. Leigh Brown, Simon D.W. Frost

**Affiliations:** 1 Department of Medicine, University of California San Diego, La Jolla, California, United States of America; 2 Department of Biochemistry, Genetics and Immunology, University of Vigo, Vigo, Spain; 3 Monogram Biosciences, South San Francisco, California, United States of America; 4 Department of Pathology, University of California San Diego, La Jolla, California, United States of America; 5 Health Protection Agency East of England Regional Epidemiology Unit, Cambridge, United Kingdom; 6 Medical Research Council Clinical Trials Unit, London, United Kingdom; 7 School of Medicine, University of Swansea, Swansea, United Kingdom; 8 Institute of Evolutionary Biology, University of Edinburgh, Edinburgh, United Kingdom; 9 Department of Veterinary Medicine, University of Cambridge, Cambridge, United Kingdom; Imperial College London, United Kingdom

## Abstract

Genetically diverse pathogens (such as Human Immunodeficiency virus type 1, HIV-1) are frequently stratified into phylogenetically or immunologically defined subtypes for classification purposes. Computational identification of such subtypes is helpful in surveillance, epidemiological analysis and detection of novel variants, e.g., circulating recombinant forms in HIV-1. A number of conceptually and technically different techniques have been proposed for determining the subtype of a query sequence, but there is not a universally optimal approach. We present a model-based phylogenetic method for automatically subtyping an HIV-1 (or other viral or bacterial) sequence, mapping the location of breakpoints and assigning parental sequences in recombinant strains as well as computing confidence levels for the inferred quantities. Our Subtype Classification Using Evolutionary ALgorithms (SCUEAL) procedure is shown to perform very well in a variety of simulation scenarios, runs in parallel when multiple sequences are being screened, and matches or exceeds the performance of existing approaches on typical empirical cases. We applied SCUEAL to all available polymerase (pol) sequences from two large databases, the Stanford Drug Resistance database and the UK HIV Drug Resistance Database. Comparing with subtypes which had previously been assigned revealed that a minor but substantial (≈5%) fraction of pure subtype sequences may in fact be within- or inter-subtype recombinants. A free implementation of SCUEAL is provided as a module for the HyPhy package and the Datamonkey web server. Our method is especially useful when an accurate automatic classification of an unknown strain is desired, and is positioned to complement and extend faster but less accurate methods. Given the increasingly frequent use of HIV subtype information in studies focusing on the effect of subtype on treatment, clinical outcome, pathogenicity and vaccine design, the importance of accurate, robust and extensible subtyping procedures is clear.

## Author Summary

There are nine different subtypes of the main group of HIV-1, each originating as a distinct subepidemic of HIV-1. The distribution of subtypes is often unique to a given geographic region of the world and constitutes a useful epidemiological and surveillance resource. The effects of viral subtype on disease progression, treatment outcome and vaccine design are being actively researched, and the importance of accurate subtyping procedures is clear. In HIV-1, subtype assignment is complicated by frequent recombination among co-circulating strains, creating new genetic mosaics or recombinant forms: 43 have been characterized to date, and many more likely exist. We present an automated phylogenetic method (SCUEAL) to accurately characterize both simple and complex HIV-1 mosaics. Using computer simulations and biological data we demonstrate that SCUEAL performs very well under various conditions, especially when some of the existing classification procedures fail. Furthermore, we show that a small, but noticeable proportion of subtype characterization stored in public databases may be incomplete or incorrect. The computational technique introduced here should provide a much more accurate characterization of HIV-1 strains, especially novel recombinants, and lead to new insights into molecular history, epidemiology and geographical distribution of the virus.

## Introduction

Many RNA viruses have evolutionary rates that hover near the mutational speed limit [1] permitting them to generate incredible sequence variability among circulating strains in a relatively short time [2]. Bottleneck events, such as viral introduction to new populations or species of hosts, followed by diversification in the new environments, create easily discernible substructures within individual viral species. For HIV-1, this substructure consists of 3 groups (M, N and O), 9 “pure” subtypes (A–D, F, G, H, J and K) of group M, and sub-subtypes (e.g. A1, A2, F1 and F2), defined entirely on the basis of phylogenetic clustering and monophyly of sequences from a given subtype in relation to all other subtypes [3]. The geographic distribution of HIV-1 subtypes is decidedly non-random [4]; for example 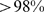 of HIV-1 circulating in North America is classified as subtype B, whereas the same subtype accounts for only 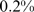 of infections in Southern Africa. This observation immediately suggests that reliable determination of viral subtypes is highly informative for epidemiological surveillance. HIV-1 diversity is sufficiently high to permit further stratification of subtypes by the geographic region of origin, yielding further clues to epidemiological history of modern epidemics [5]. However, because several established subtypes often circulate concurrently in one host population [6], and because HIV has exceptionally high recombination rates [7], novel recombinant forms are frequently generated. If at least three epidemiologically unrelated viral isolates show an identical novel recombination structure in terms of the pure subtype reference strains, a new circulating recombinant form (CRF) is added to the compendium maintained by the Los Alamos National Laboratory (http://www.hiv.lanl.gov/content/sequence/HIV/CRFs/CRFs.html). There are currently 43 described CRFs, differing widely in their prevalence, range and the complexity of the recombinant structure. However, the relationship between CRFs and their parental strains is not always clear cut; for example CRF02, originally thought to have been the product of recombination between subtype A and subtype G strains could in fact be ancestral to subtype G strains [8].

A number of computational approaches have been proposed to classify viral strains into subtypes or to describe recombinant strains as mosaics of subtypes. Unlike with methods geared towards a more general problem of detecting recombination from sequence alignments [9],[10], there are no comprehensive comparative benchmarking studies for subtyping methods in the literature. The methods can be conceptually categorized by whether or not they explicitly use a phylogeny to assign subtypes, whether or not they require a multiple sequence alignment and by the degree of automation that they afford: full, partial or none. The de facto standard for accurately describing novel recombinant forms has changed little since its introduction in [11]. It consists of an initial sliding-window phylogenetic bootstrap (bootscanning) analysis of the query sequence aligned against the set reference strain used to generate the set of apparent breakpoints which are then confirmed by detailed phylogenetic analysis of putative non-recombinant fragments. This is a powerful and intuitively attractive, but laborious method–the entire process frequently lacks automation (e.g. [12],[13], but see [14]), has many user-adjustable parameters, such as the alignment procedure, reference sequences, sliding window size and stride, precise location of breakpoints, phylogenetic bootstrap values that are selected subjectively, and can lead to ambiguous or not fully resolved results (e.g. [15] vs [16], [17]). Perhaps the single greatest criticism of the bootscan/phylogeny approach may be that two alternative characterizations of the same query sequence are not assigned a statistically meaningful goodness-of-fit score, and hence cannot be objectively compared.

On the other end of the spectrum are fully automated techniques, including a sophisticated phylogeny and alignment based REGA v2.0 tool [18], henceforth referred to as REGA, and several phylogeny and/or alignment free tools: a classification method based on subtype-specific distributions of short nucleotide strings [19]; a sliding window analysis based on BLAST scores of the query and each of the subtype reference sequences [20]; a phylogeny free position/subtype specific amino-acid subtype analyzer (STAR) which assigns each residue in a multiple sequence alignment a subtype discriminating score [21]; and a probabilistic jumping alignment approach jpHMM [22] that uses a hidden Markov model to align the query to the locally most similar reference sequence.

Alignment and/or phylogeny free techniques are fundamentally approximate in nature, because the *definition* of a subtype is rooted in the concept of a clade and hence is intrinsically phylogenetic in nature. Approximate approaches have been developed to address the very practical issues of automation, speed and the fact that a phylogenetic definition of a subtype becomes complicated when reference strains are permitted to have recombined themselves. On the other hand, these methods often produce conflicting or indeterminate results, may be unable to classify novel or rare mosaics, and frequently disagree with manually performed phylogenetic analyses, causing considerable consternation among practitioners and clinicians (e.g. [23]–[25]). A recent comparative study of three automated subtyping tools on 10537 partial polymerase sequences from the UK [26] found that methods agreed poorly (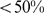) for subtypes other than B,C and H, failed to classify 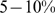 of sequences and returned discordant results in 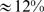 cases of divergent sequences, which were revealed to be unusual recombinant forms by a laborious follow-up analysis.

Hence, we are convinced that it is necessary to adopt a phylogeny-based method for accurate subtyping. Statistical evidence of phylogenetic incongruence, i.e. instances when different regions of an alignment support discordant phylogenies, is a hallmark of recombination [27]. A statistically robust phylogenetic approach to detecting phylogenetic incongruence in a multiple sequence alignment has been proposed in the Bayesian framework by [28] and in the information theory framework by [29]. These methods are powerful but too slow to be practical for large reference phylogenies needed to describe extant HIV diversity–for example our HIV-1 polymerase reference alignment contains nearly 300 sequences. Because subtyping is a particular case of more general recombination analyses, we devised an algorithm whose run time is effectively constant in the size of the reference alignment. Importantly, this is achieved without collapsing the alignment into a collection of attributes, such as substring frequencies or position-specific alignment scoring matrices, as is frequently done by phylogeny-free methods.

Our design objectives for SCUEAL included: (i) a completely automatic method, which returns a predicted subtype, existing CRF or a recombinant form mapped in terms of the former; (ii) every estimated quantity including the recombinant structure, the location of each breakpoint and the assignment of a parental/sister lineage should be estimated with statistical confidence/support values to allow an objective evaluation of how robust the estimates are; (iii) the algorithm runs sufficiently quickly (2–3 CPU minutes to screen a simple sequence, and up to a CPU hour for highly complex mosaics) to permit the screening of thousands of sequences on a computer cluster. We implemented an easy-to-use web interface to SCUEAL running on the datamonkey.org [30] platform); (iv) accepts large reference sequence alignments which can be easily updated when new references (e.g. CRF) become available. SCUEAL is conceptually based on the more general method (GARD) for detecting recombination in multiple sequence alignments presented in [29], but is an entirely new algorithm and software implementation. Whereas GARD is primarily concerned with detecting the number and location of breakpoints in an alignment, and not in identifying recombinant lineages and clades (which is critically important for subtyping), SCUEAL explicitly searches for both using a significantly modified and improved genetic algorithm. Also, by screening a single sequence against a fixed reference alignment, SCUEAL gains significant power and an order of magnitude speed-up over GARD, which assumes that any sequence can be a recombinant.

We assessed various performance metrics of SCUEAL using an extensive set of simulations and biological data; to our knowledge no other method has been subjected to a comparably exhaustive benchmarking study.

## Methods

Consider an alignment of 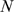 reference sequences on 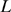 bases, each labeled with its subtype. We require that none of the reference sequences have undergone detectable recombination, hence their evolutionary history can be accurately described with a single phylogenetic tree, 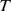; note that this framework can be used to handle recombinant reference sequences represented as multiple partial sequences (see below). In this manuscript, the evolution of extant sequences from their most recent common ancestor along the phylogenetic tree is described by the general time reversible model of nucleotide substitution [31] and site-to-site rate variation is accommodated via a 3-bin general discrete distribution (e.g. [32]). Substitution models for codon and protein evolution can be easily accommodated by the testing framework; however because they incur considerable additional computational expense they are not considered here.

### Phylogenetic mosaics

The objective of our methodology is to enable automatic identification of the number (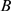) and location of any recombination breakpoints in a query sequence, that is assumed to be homologous and alignable to the reference sequences, together with the identities of sister lineages in each non-recombinant fragment. An example of such an assignment can be found in Figure 0: the query sequence (labeled Q) has two recombination breakpoints, at nucleotide positions 750 and 1250. Over the first 750 nucleotides, the query sequence shares a common ancestor with reference sequence 1, over the next 500 nucleotides - with reference sequence 7, and over the last 750 nucleotides - with sequence 1 again. Such an arrangement might arise if the query is the result of a recombination event between the ancestors of sequences 1 and 7.

The term ‘mosaic’ has come to encompass the combination of breakpoint placements and lineage assignments in HIV-1 subtyping literature. The number of possible mosaics with 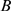 breakpoints is proportional to 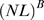, hence it is not practical to undertake an exhaustive search of all possible mosaics, unless 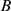 is small (*i.e.* B = 1 or B = 2).

### Model fitting and fitness evaluation

In order to select credible mosaics from the set of all possible models we must be able to compute a goodness-of-fit value for each proposed mosaic.

We begin by computing the maximum likelihood based score for each model. First, we fit the reference tree to the reference alignment using standard phylogenetic maximum likelihood. Assuming unrooted bifurcating trees, 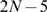 branch length estimates and 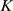 substitution model estimates, such as relative nucleotide substitution rates, base frequencies and site-to-site rate variation parameters will be obtained. These *baseline* parameters are estimated once for a reference alignment, and can be reused if multiple query sequences are run against the same reference.

**Figure 1:**
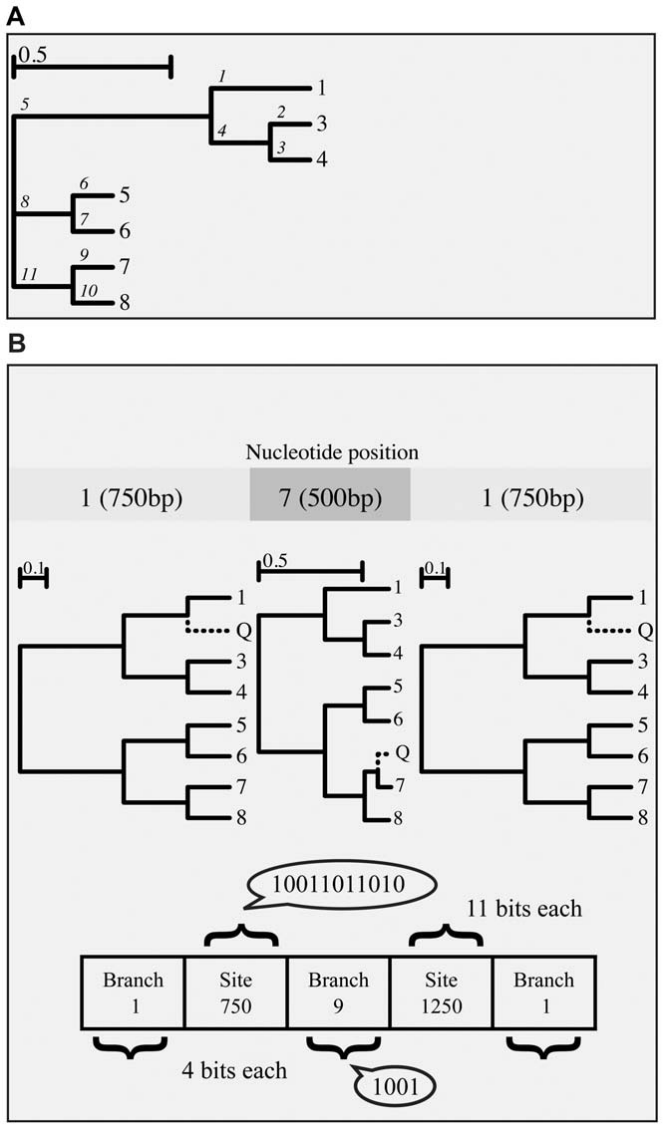
An example to illustrate the concepts of a mosaic and its binary encoding upon which the genetic algorithm operates. Panel A: a phylogenetic breakpoint/lineage model which “threads” a query sequence (labeled ‘Q’) onto the reference tree with 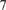 sequences. Panel B: the example individual model (mosaic) 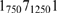 is encoded by a 36-bit binary vector on 5 fragments (genes)–2 for placing the breakpoints (Gray-binary encoded) and 3 for identifying sister lineages, binary encoded using the post-order traversal scheme shown in the reference tree of Panel A.

For computational efficiency we fix all substitution model parameters at their baseline values instead of re-estimating them for each mosaic. If the reference alignment is sufficiently large, the effect of one additional sequence on substitution model parameters will be insignificant. Furthermore, we posit that grafting the query sequence onto a branch in the reference tree will only affect three branch lengths for each non-recombinant fragment. For instance, for the mosaic shown in Figure 1 the algorithm will estimate three branch lengths for the first segment (those leading to 1 and Q as well as the branch leading to their MRCA), three branch lengths of the second segment (Q,7 and the MRCA of Q and 7) and three branch lengths for the third segment (1,Q and the MRCA of 1 and Q). All other branch lengths are maintained at the values derived from the reference tree. Similar approximations are routinely made in phylogenetic inference (e.g. [33],[34]). The fitness of mosaic 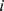 is evaluated using Schwartz's Bayesian Information Criterion (BIC, [35]), with the number of model parameters for a mosaic with 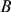 breakpoints given by 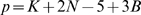: 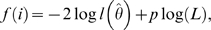
(1)where 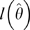 is the likelihood of the data under the mosaic model maximized over 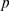 parameters and 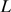 is the number of sites in the alignment, used to approximate the number of independent observations. A lower BIC score indicates a better fit to the data. BIC was selected because it had the best power/accuracy performance in our initial simulation studies, comparing AIC [36], AIC-c [37] and BIC (results not shown).

The immediate benefit of allowing only three branch lengths to vary per segment is that the computational cost for fitting individual mosaics no longer depends on the size of the reference alignment, at least when time-reversible models of substitutions are used. This observation has been exploited in many phylogenetic applications and is discussed in detail for example in [38]. Briefly, as a part of standard phylogenetic likelihood evaluation [39], each node 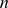 (both tips and internal nodes) of the phylogenetic tree is populated with a vector of partial probabilities 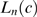 that containts the probability of observing the subtree rooted at 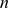 if the character (i.e. a nucleotide in our case) at 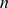 is 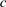. To evaluate the likelihood of the entire tree (for a single site), the following expression is computed at the root: 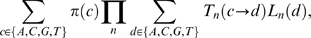
where 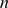 iterates over the children of the root node, 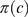 gives the stationary frequency of nucleotide 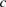 (estimated by counts from the data) and 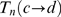 denotes the probability of substituting nucleotide 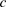 with nucleotide 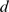 along the branch that ends in 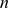. The critical observation to be made here is that if nothing but the lengths of branch emanating from the root node change during optimization (i.e. only 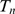 changes), then 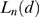 do not have to be recomputed, reducing the complexity optimization problem to that on a star tree with 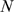
( = 3 for standard phylogenetic applications) tips.

For time-reversible models, the root can be arbitrarily placed on any branch of the phylogenetic tree. Hence, we can reroot the tree at the point where the query sequence is grafted and reduce the computational complexity as explained above. To do this, in addition to 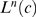, we also precompute (for every node except the root and only once per analysis) the collection of vectors 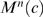, that contain conditional probabilities of the *parent* node of 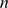, when 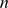 is considered as the root node. For every non-root node 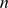 the likelihood of the bifurcating reference tree can be equivalently expressed as: 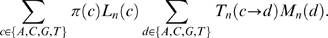
The last expression is simply the likelihood of the tree rerooted exactly at node 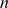. Grafting the query sequence 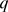 onto the branch leading to node 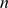 will create three branches: the branch leading to 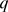, the branch leading to 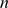 and the branch leading from the ancestor of 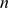 and 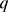 (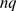) to the parent of 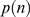 For the first partition in Figure 1, for example, the single branch of the reference tree leading to tip 1, was transformed into three branches by grafting Q–the branch leading to tip 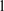, the branch leading to query 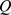 and the branch leading to the parent of 1 and Q. Consequently, the likelihood of the tree with the query sequence 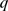 grafted onto the branch leading to 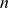 can be computed as:
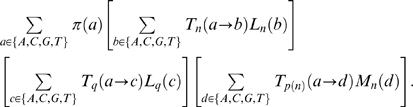
This expression is the likelihood of a three-taxon star tree with the root at node 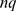 (sum over 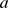) and three children: 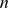 (sum over 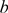), 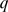 (sum over 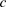) and the parent of 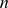, 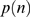 (sum over 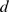). Note that because 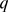 is always a tip, the conditional probabilities in 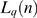 are trivial to compute, and it follows that the cost of evaluating the likelihood of the reference tree with a grafted tip (given precomputed quantities, 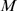 and 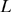
–done only once for the reference alignment, independent of the query sequence) is equivalent to the three-taxon case.

### Mosaic selection using a genetic algorithm

We use an aggressive genetic algorithm (GA) with elitist selection that is based on the CHC procedure [40] to rapidly search a combinatorially large space of possible mosaics for a fixed number of breakpoints. The algorithm operates on a population of 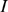 binary strings (individuals), each representing an encoded mosaic with 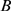 breakpoints. 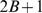 fragments (‘genes’) are needed to encode the mosaic - 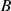 for the location of breakpoints, and 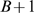 for lineage assignments on each non-recombinant fragment (see Figure 1). We restrict breakpoints to only occur at variable alignment sites as was done previously in our GARD method [29]. In addition, the breakpoints must be a minimum distance (denoted as a tunable parameter 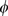) away from each other or from the ends of the sequence; this simply reflects the fact that a minimum number of sites is necessary to resolve the phylogenetic placement of a sequence.

The placement of the query sequence in the reference tree is represented by the binary-encoded position of the branch in post-order traversal (cf. Figure 1). Breakpoint positions are represented using Gray binary coding, to ensure any two consecutive locations differ by a single bit, and hence can be reached by a single mutation [41]. For example, to change the position of a breakpoint from 7 (traditional binary 0111, Gray code 0100) to 8 (1000; 1100) it would be necessary to mutate all four bits in the traditional binary code, but only one bit in the Gray code. Breakpoints are always maintained in left-to-right ordering and any operations that disrupt this order are followed by resorting of breakpoints left to right (equivalent to gene order rearrangement).

**Figure 2:**
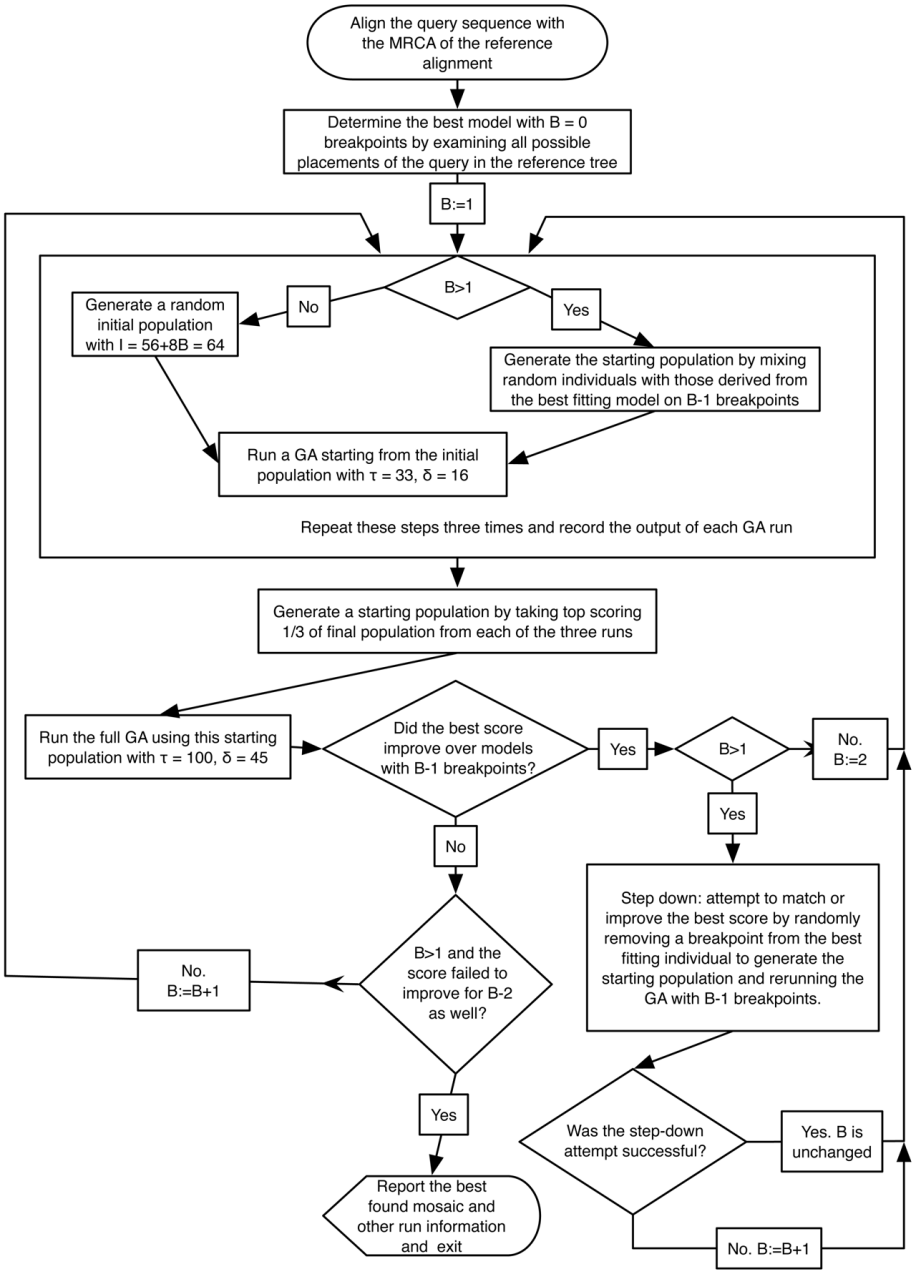
Algorithmic flowchart of SCUEAL. Algorithmic logic underlying SCUEAL; see Figure 3 for a description of the genetic algorithm itself. Refer to the text for more detailed descriptions of individual procedures and parameter definitions.

**Figure 3:**
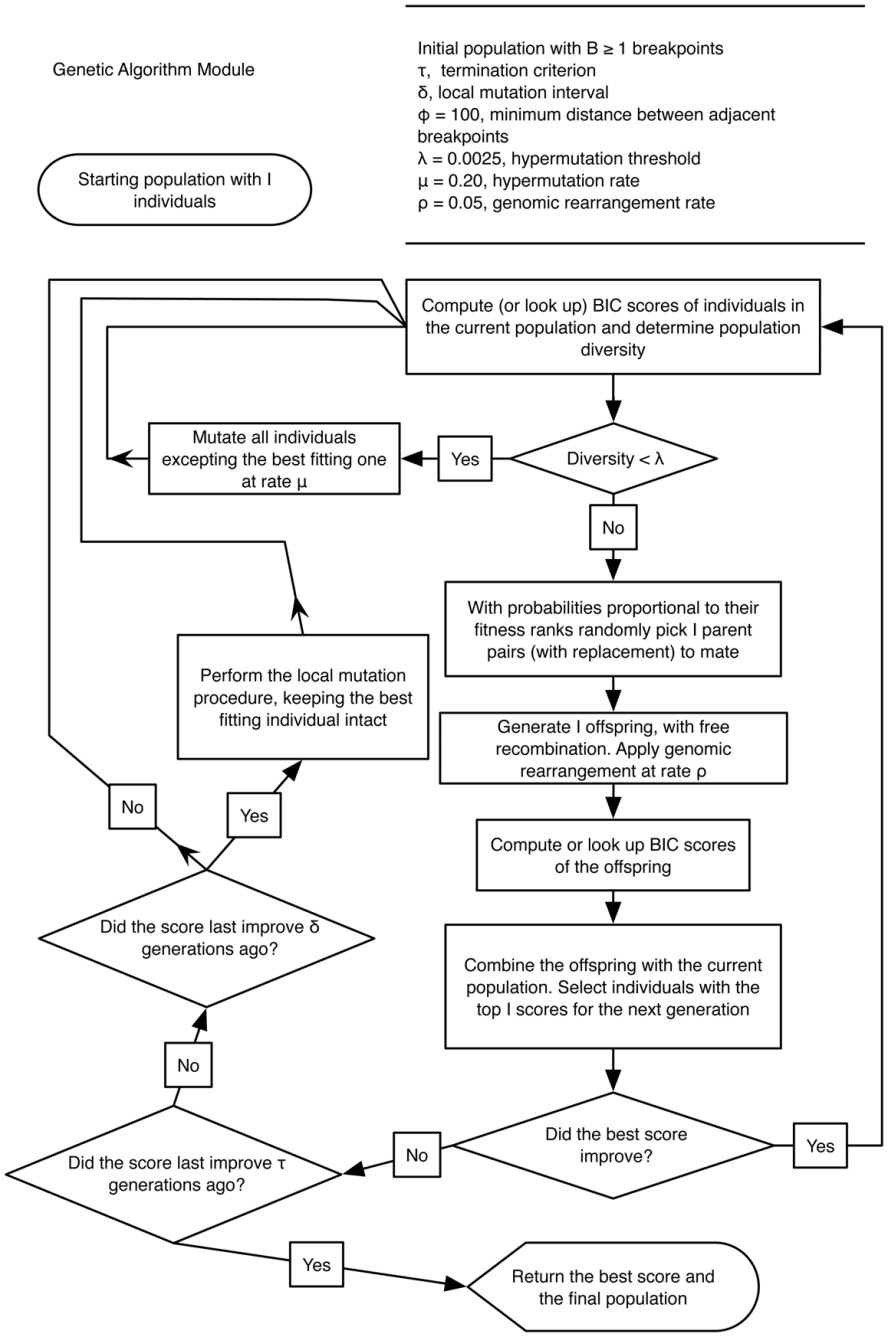
Algorithmic flowchart of the genetic algorithm in SCUEAL. A flowchart description of the genetic algorithm applied to a given starting population and controlled by input parameter values. Refer to the text and Figure 2 for further description of individual steps and parameter definitions.

Starting with the initial population of 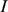 mosaics, the algorithm proceeds as follows (refer to Figures 2 and 3 for a graphical description of the procedure). First, fitness of each mosaic 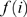 (Eq. 1) is computed and the mosiac is assigned a mating probability inversely proportional to its fitness rank 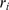. The most fit mosaic reproduces becomes a parent for an offspring with 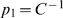, while the least fit mosaic–with probability 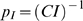, where 
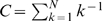
. The algorithm maintains a global lookup table (implemented as an AVL tree keyed on the bit string of the mosaic) to ensure that the maximum likelihood fitting of any given mosaic is carried out only once. Second, 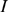 pairs of parents are selected based on their mating probabilities to generate 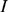 offspring. The mating operator uses free recombination, where every bit of the child has a 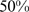 probability of coming from either parent; this ensures rapid mixing of mosaic features. With probability 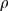 the algorithm also induces genomic rearrangement in the offspring mosaic, by swapping adjacent fragments around a randomly selected breakpoint. Third, the existing population is augmented with the offspring, resulting in 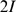 mosaics, ranked according to BIC and filtered to include 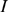 top-scoring mosaics in the next generation; this induces a strong selective pressure to remove mosaics with low fitness scores.

Mutational processes are available to re-introduce genetic variability into inbred populations. First, hypermutation is triggered if the diversity of the population, measured as the relative difference between in BIC between the best and worst fitting mosaic (i.e. 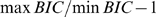), drops below a fixed threshold, 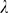. All mosaics in the population, except the best fitting one, have their bits toggled with fixed probability 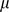. Second, if no generation-to-generation BIC improvement was observed for 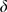 consecutive generations, local mutation is carried out. The bottom two thirds of the population are replaced by mutated versions of the best fitting mosaic, generated by selecting a fragment to mutate at random and providing local coverage for that fragment. Local coverage is introduced by first drawing a random branch if the gene encodes a lineage, or a random position within 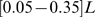 bp of the current position for a breakpoint location gene, and then generating 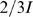 consecutive values for the gene. For example, if the new random position for the breakpoint is drawn as 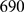, then mosaics with the breakpoint at 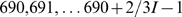 will be placed in the population.

The algorithm terminates if no BIC improvement has been obtained for 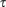 consecutive generations. The number of breakpoints is increased from 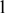 until no BIC improvement has been found for two consecutive values of 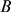. The case of 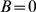 is solved exhaustively; the initial population for 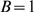 is generated randomly; the initial population for 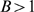 is seeded by the best mosaic from the 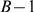 run, with a randomly placed additional breakpoint and lineage assignment. For 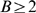, we also add a step down procedure to confirm that the improvement in score obtained by incrementing 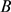 was due to a genuine additional breakpoint and not due to premature termination at the previous step (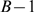); to do so, we generate 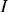 mosaics by randomly removing a breakpoint from the best-fitting mosaic with 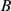 breakpoints (randomly assigning the query sequence to one of the two parental lineages, and introducing mutations at rate 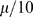) and run an iteration of the GA with 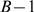 points using the 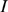 mosaics as a starting population. If the follow-up GA with 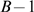 breakpoints matches or improves upon the score with 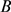 breakpoints, then the next phase of the GA is run on 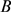 breakpoints, otherwise, the next phase operates on 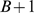 breakpoints. To further enhance algorithm robustness, we evolve three independent populations (from completely random starting mosaics) to convergence, compose the mixed population by taking the top-scoring third of each population and evolve the combined population until convergence.

While it is possible to use the GA to also search for 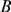 directly (e.g. by duplicating or removing fragments), we found that the incremental search for 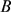 with the step-down verification stage has better convergence properties and runs considerably faster.

Algorithm parameter values selected for the analyses in this paper are as follows: 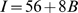, 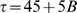, 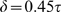, 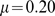, 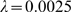, 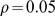, 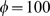. parameter values were selected based on our previous experience with GARD [29], and further adjusted based on how well the algorithm performed on simulated data and run time.

### Result processing

After a GA run, BIC scores and mosaics from a large (typically 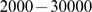) number (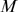) of fitted mosaics is available for processing. Instead of basing inference on the single best fitting mosaic, we adopt a multi-model inference procedure, whereby the contribution of each fitted mosaic is weighted based on its goodness-of-fit. Given the BIC score (fitness) of the best mosaic from the run, 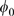, for every mosaic 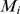, we compute its Akaike weight, 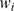 defined in terms of its BIC score 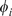 as 
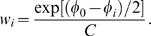
The constant 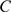 is chosen so that 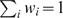. 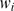 can be interpreted as the probability that the *i*-th mosaic provides the best fit to the data [42].

We report the following quantities for each GA mosaic screen

1. The structure of the best fitting mosaic, represented as the location of inferred breakpoints and lineage assignments, e.g. 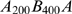.
2. The model averaged support for the mosaic structure of the best model. This is defined as the sum of Akaike weights of all those models which agree with the best fitting model in everything except the coordinates of the breakpoints. E.g. 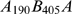 is consistent with 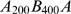, but 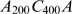 is not. High values (e.g. 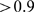) of the model averaged support indicate that there are no other discordant mosaic structures that explain the evolutionary history of the query sequence.
3. Model-averaged support for the locations of the breakpoints, that is computed by tabulating the model-averaged probability of observing a breakpoint at a given site over all sites in the alignment, based on the normalized Akaike weights of the models whose mosaics are consistent with the best fitting model. For instance the model 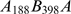 will contribute its Akaike weight to sites 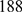 and 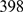. To determine 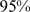 confidence intervals for each breakpoint from the best fitting model, we build symmetric intervals around each breakpoint that contain at least 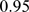 the support. Note that confidence intervals are not uniquely defined in this setting (for example, we could extend the interval in the direction where the site immediately outside the current interval has greater model averaged support of a breakpoint), and we adopt symmetric intervals for simplicity.

### Automated sequence alignment

The genetic algorithm requires the alignment of reference sequences with the query sequence as input that can be generated by any of the multiple sequence alignment programs. However because the reference alignment does not depend on the query sequence, it does not need to be re-aligned every time a new sequence is queried against it and the following simple heuristic can be employed. We preprocess the reference alignment by fitting an evolutionary model (nucleotide or codon for coding alignments) using the reference tree and inferring the root sequence for the reference tree using the joint maximum likelihood of [43]. Gaps in the alignment are treated as missing data from the purposes of root sequence reconstruction. In particular, the root sequence will not contain any gaps when reconstructed under standard nucleotide evolutionary models, because no sites in the reference alignment consist solely of gaps. This inferred root sequence can then be directly aligned with the query sequence using the Needleman-Wunsch dynamic programming algorithm [44], with affine gap costs and zero prefix and suffix gap costs on nucleotide or translated amino-acid data, and then up-converted into a multiple sequence alignment with all reference sequences consistent with the reference alignment. When aligning HIV or other viral sequences, organism specific scoring matrices [45] can be used to improve alignment quality. In addition to being very fast, this alignment heuristic is unlikely to introduce difficult-to-quantify biases common in progressive alignment approaches (e.g. [46]).

### Reference alignment generation

We adopted a step-wise procedure of HIV-1 reference alignment construction. Beginning with a seed alignment of three sequences (e.g. one each from A, B and C for HIV-1), screened by GARD to ensure that the seed sequences are not recombinant, we augment the seed alignment from a collection of potential subtype reference sequences downloaded from the LANL HIV database. If a database sequence is labelled as pure subtype in LANL, is at least 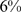 distant (Tamura-Nei 93 [47] genetic distance) from every sequence in the seed alignment, and is reported as being non-recombinant by SCUEAL, then it is added to the reference alignment. The process repeats until the collection of potential reference sequences has been exhausted.

Reference sequences for circulating recombinant forms (CRFs) are processed in a similar way, except that if the CRF sequence has 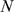 breakpoints in the region for which the reference alignment is being built (e.g. the pol gene), then it is represented by up to 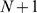 sequences in the final alignment. For instance, a 1000 bp sequence with the mosaic structure 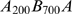, will be represented by a sequence that clusters with the A clade and contains bases from 1–199 and 700–1000 and gaps between positions 200 and 699, and a complementary sequence (bases between 200 and 699, gaps elsewhere) that clusters with clade B. This is necessary to correctly place a recombinant sequence on the single reference tree. The GA disallows mosaic structures in which a query sequence would cluster with artificially introduced gaps in CRF component sequences. SCUEAL will correctly interpret clustering with the constituent sequences as clustering with the single CRF for the purposes of subsequent inference.

The resulting full length HIV-1 reference polymerase alignment comprised 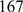 sequences encompassing the “pure” subtypes (including 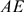, 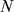 and 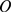 clades), SIVcpz and a reference strain from each of the CRFs (except CRF26,CRF38 and CRF41–43) for which no full length pol reference sequences were present in the database) listed in the Los Alamos HIV CRF compendium (http://www.hiv.lanl.gov/content/sequence/HIV/CRFs/CRFs.html accessed December 17th, 2008). We note that this procedure is not guaranteed to avoid mislabeling recombinant sequences as pure subtypes. Indeed if the recombinant strain is added to the reference before the parental strains, the latter will be incorrectly described as recombinants. For example, the original classification of subtype G sequences as a “pure” subtype is likely an artifact of the order in which A,G and CRF02 sequences were added to public databases [8]. Nonetheless, our procedure is undoubtedly an improvement over simply taking a collection of database sequences as a reference and assuming that they can be adequately described by a single tree; this practice should be avoided.

### Simulated data

**Figure 4:**
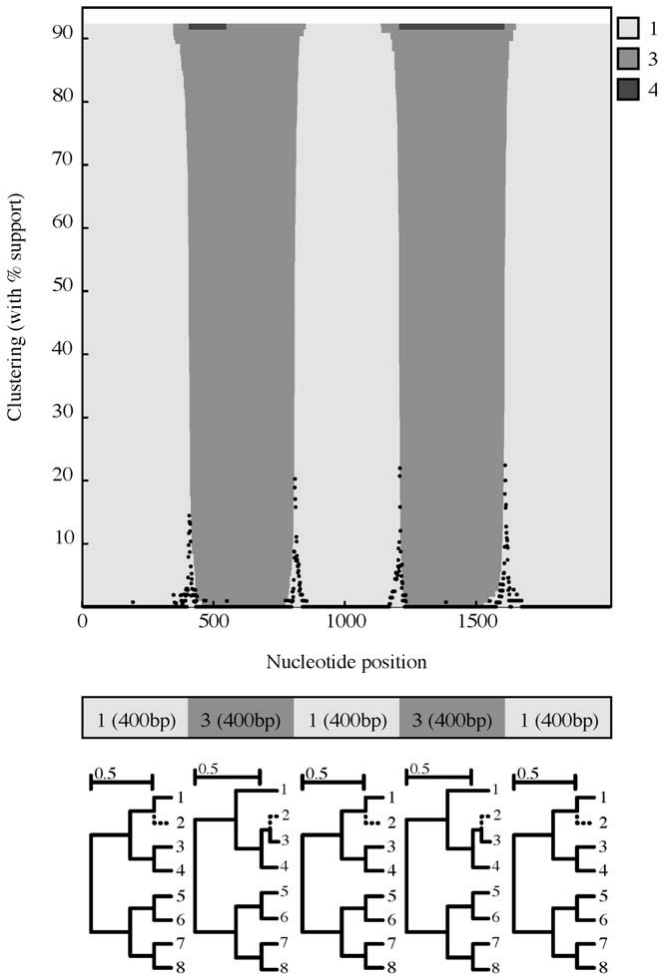
A simulation scenario example. One of the simulation scenarios used to asses our detection method with the results over 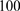 replicates (scenario 5/close in Table 2). The query sequence (2) was simulated to move from reference lineage 1 to reference lineage 3 every 400 bp as shown in the tree panel. The clustering chart depicts model and replicate averaged support for assigning the query sequence to a particular reference lineage, as estimated by the genetic algorithm over 100 simulated data replicates, whereas black impulse plots indicate the inferred placements of breakpoints. The y-axis does not reach 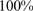 because each replicate contributes the model averaged support for the best inferred mosaic type–a value that is 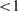; the upper limit on the y-axis is, therefore, the mean (over replicates) model-averaged support for the best-fitting mosaic (0.92 in this case).

Each of the simulation scenarios summarized in Table 1 comprised 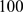 parametrically generated alignments, using the general time reversible model of nucleotide substitution [31], equilibrium base frequencies of 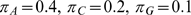 and 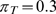, substitution rate parameters of 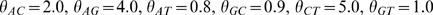, and site-to-site rate heterogeneity modeled a G+I distribution with 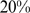 of invariant sites and the shape parameter of 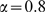; all these parameters were selected to resemble values found in biological alignments of HIV-1. Recombination was introduced by generating alignments of fixed lengths along different tree topologies and then concatenating them; the spacing between breakpoints, tree topologies used and recombinant lineages are shown in the middle pane of each each figure; simulated data are available at http://www.hyphy.org/pubs/SCUEAL/. The trees were constrained to conform to the assumptions of the model–only one sequence (the query) was permitted to migrate from lineage to lineage. The correct tree and reference sequences were used for screening. The evolutionary scenarios used for simulation were designed to cover a range of recombination patterns with respect to the distribution of breakpoints, the level of sequence divergence and how far in the tree the recombinant sequence moved (close, medium or divergent). A subset of scenarios dealt with ‘ancient’ recombination events, i.e. lineage assignments to internal tree branches (for HIV-1 this would be equivalent to the recombination event predating the proliferation of the subtype). Several examples were specifically selected to mimic different divergence levels of HIV-1. An example of one recombination scenario is given in Figure 4 and the number and location of breakpoints can be found in Table 1. The collection of analogous figures for every simulation scenario can be found in Protocol S1.

**Table 1:** SCUEAL performance on simulated data.

Scenario	Seq., sites	Type/Distance	Inferred Mosaics		Breakpoints	
			Type	Count ( 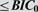 )	Simulated Location, Parents	Inferred. #/Median Location Std.Dev. (95% Range)
1. No recombination	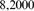	N/A	Correct	100 (100)	None	
2. An evident breakpoint	8,2000	Close ( 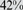 )	Correct	100 (88)	1000 bp 1∶3	100/990,18.99 (931,1015)
		Divergent ( 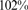 )	Correct	100 (75)	1000 bp 1∶7	100/1000, 5.04 (987,1007)
		Ancient ( 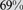 )	Correct	92 (86)	1000 bp 1/2∶5/6	96/992,16.62 (947,1017)
			Superset	7 (6)		
			M/M	1 (1)		
3. Two evident breakpoints	8,2000	Close ( 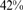 )	Correct	98 (96)	750 bp 1∶3	99/749,10.23 (720,769)
		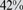	Superset	1 (1)	1250 bp 7∶1	99/1251,15.26 (1201,1273)
			M/M	1 (1)		
		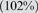 Divergent	Correct	95 (89)	750 bp 1∶7	98/751, 5.61 (735,762)
		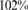	Superset	5 (5)	1250 bp 7∶1	100/1251, 5.92 (1237,1265)
		Ancient (69%)	Correct	91 (90)	750 bp 1/2∶5/6	96/749,22.37 (697,824)
		69%	Superset	5 (4)	1250 bp 5/6∶1/2	96/1250,20.09 (1192,1283)
			M/M	4 (4)		
4. Two close breakpoints	8, 2000	Close (42%)	Correct	22 (21)	950 bp 1∶3	22/948,15.35 (888,960)
		42%	Subset	77 (76)	1050 bp 3∶1	22/1050, 7.77 (1031,1066)
			M/M	1 (1)		
		Divergent (102%)	Correct	73 (69)	950 bp 1∶7	73/951, 6.40 (932,960)
		102%	Subset	11 (0)	1050 bp 7∶1	73/1051, 5.79 (1038,1068)
			M/M	16 (15)		
5. Four breakpoints	8, 2000	Close (42%)	Correct	96 (96)	400 bp 1∶3	98/399,16.86 (342,428)
		42%	Superset	3 (2)	800 bp 3∶1	97/803, 9.63 (784,837)
		42%	M/M	1 (1)	1200 bp 1∶3	98/1200,11.34 (1161,1220)
		42%			1600 bp 3∶1	99/1602,12.24 (1570,1634)
		Divergent (102%)	Correct	96 (96)	400 bp 1∶7	98/401, 5.08 (389,413)
		102%	Superset	2 (2)	800 bp 7∶1	99/802, 5.00 (785,809)
		102%	M/M	2 (2)	1200 bp 1∶7	99/1201, 5.73 (1188,1211)
		102%			1600 bp 7∶1	99/1602, 4.19 (1594,1613)
		Ancient (69%)	Correct	54 (54)	400 bp 1/2∶5/6	65/402,14.88 (357,434)
		69%	Subset	22 (3)	800 bp 5/6∶1/2	67/802,15.74 (745,826)
		69%	M/M	20 (19)	1200 bp 1/2∶5/6	69/1201,19.67 (1169,1270)
		69%	Superset	4 (4)	1600 bp 5/6∶1/2	69/1602,18.04 (1550,1627)
6. Nine breakpoints	8,2000	Close (42%)	Correct	30 (30)	200 bp 1∶3	68/201,11.72 (176,235)
		42%	Subset	13 (2)	400 bp 3∶1	62/403, 8.18 (391,420))
		42%	Superset	9 (7)	600 bp 1∶3	68/601,14.69 (561,634)
		42%		48 (25)	800 bp 3∶1	71/803,10.72 (783,838)
		42%			1000 bp 1∶3	71/1001,10.95 (974,1021)
		42%			1200 bp 3∶1	72/1203,13.14 (1177,1253)
		42%			1400 bp 1∶3	75/1401,11.45 (1364,1414)
		42%			1600 bp 3∶1	73/1602, 8.52 (1582,1626)
		42%			1800 bp 1∶3	77/1801,13.40 (1746,1816)
		Divergent	(102%) Correct	64 (64)	200 bp 1∶7	96/202, 4.87 (188,212)
		102%	Superset	9(7)	400 bp 7∶1	94/402, 7.98 (386,415)
		102%	M/M	27 (25)	600 bp 1∶7	93/601, 5.95 (591,625)
		102%			800 bp 7∶1	93/802, 5.37 (790,815)
		102%			1000 bp 1∶7	92/1002, 5.35 (985,1015)
		102%			1200 bp 7∶1	93/1202, 6.17 (1191,1228)
		102%			1400 bp 1∶7	93/1402, 4.52 (1391,1411)
		102%			1600 bp 7∶1	93/1602, 4.05 (1594,1612)
		102%			1800 bp 1∶7	89/1802, 3.80 (1794,1814)
7. Complex mosaic	8, 2000	42%	Correct	88 (86)	400 bp 1∶2	94/400,11.89 (375,440)
		12%	Subset	3 (1)	800 bp 3∶4	89/793,28.29 (737,853)
		108%	Superset	5 (4)	1200 bp 4∶7	98/1202, 4.02 (1192,1211)
		48%	M/M	4(4)	1600 bp 7∶5	98/1601.5,11.08 (1586,1640)
8. HIV within-patient	13, 2000	Close (0.4%)	Subset	96 (96)	750 bp 1∶2	
		0.4%	M/M	4 (4)	1250 bp 2∶1	
		Divergent (2.3%)	Correct	38 (36)	750 bp 1∶9	38/741.5,34.62 (666,790)
		2.3%	Subset	4 (2)	1250 bp 9∶1	39/1256,36.11 (1156,1326)
			Superset	1 (1)		
			M/M	57 (55)		
9. HIV within-patient	13, 2000	Close (0.4%)	Subset	97 (97)	400 bp 1∶2	
		0.4%	M/M	3 (3)	800 bp 2∶1	
		0.4%			1200 bp 1∶2	
		0.4%			1650 bp 2∶1	
		Divergent (2.9%)	Correct	7 (7)	400 bp 1∶9	16/391.5,32.00 (349,475)
		2.9%	Subset	2 (1)	800 bp 9∶1	21/808,39.70 (730,868)
		2.9%	Superset	1 (0)	1200 bp 1∶9	22/1202.5,42.90 (1118,1284)
		2.9%	M/M	90 (70)	1600 bp 9∶1	20/1610.5,32.87 (1551,1676)
10. HIV within-subtype	5, 2000	4%	Correct	16 (16)	400 bp 1∶2	30/402,31.53 (317,460)
		4%	Subset	80 (77)	800 bp 2∶1	21/802,35.56 (716,885)
		4%	M/M	4 (4)	1200 bp 1∶2	20/1209,30.18 (1151,1266)
		4%			1600 bp 2∶1	35/1589,38.80 (1506,1689)
11. HIV mosaic	12, 10000	Close (12%)	Correct	95 (95)	2000 bp 1∶2	94/2002.5,30.11 (1925,2092)
		12%	Subset	1 (0)	4000 bp 2∶1	93/4000,29.62 (3928,4085)
		12%	Superset	2(2)	6000 bp 1∶2	94/6002.5,26.99 (5941,6067)
		12%	M/M	2(2)	8000 bp 2∶1	92/7996,33.39 (7929,8078)
		Intermediate (12%)	Correct	100 (100)	2000 bp 1∶6	100/2000,17.40 (1959,2042)
		12%			4000 bp 6∶1	99/4003,21.62 (3964,4053)
		12%			6000 bp 1∶6	100/6001,18.61 (5952,6040))
		12%			8000 bp 6∶1	99/8004,16.88 (7968,8046)
		Divergent (11.5%)	Correct	99 (97)	2000 bp 1∶9	99/2002,20.92 (1956,2043)
		11.5%	Superset	1 (1)	4000 bp 9∶1	100/4002.5,19.85 (3945,4056)
		11.5%			6000 bp 1∶9	98/6000,21.89 (5937,6042)
		11.5%			8000 bp 9∶1	99/7999,22.49 (7953,8070)
		Complex 12%	Correct	94 (93)	2000 bp 1∶2	96/2003,27.61 (1940,2070)
		14%	Superset	5 (4)	4000 bp 2∶6	99/4000,18.14 (3969,4053)
		12%	M/M	1 (1)	6000 bp 6∶1	100/6003,20.35 (5959,6068)
		11.5%			8000 bp 1∶9	97/8000,21.34 (7947,8062)

**Scenario provides a brief description a given simulation scenario. Seq., sites** lists the number and length of simulated sequences. **Type/distance** classifies the simulation scenario by type and mean divergence between parental strains, measured as the total branch length (expected number of substitutions/site 100%) between the strains. **Inferred Mosaics** tabulates the number of cases (and the number of those that matched or bested the BIC score of the correct model) that fell into each of the classification categories (see main text for further detail). Correct: the simulated mosaic was recovered; superset: the simulated mosaic and superfluous breakpoints were inferred; subset: a partial correct mosaic was recapitulated (some breakpoints missing); and M/M - the inferred mosaic was a mismatch with the generating one. **Breakpoints** enumerates the location of each simulated breakpoint and its parental lineages, the number of times the breakpoint was recovered by SCUEAL, and the median (2.5%–97.5% range) of the distribution of distances between the simulated and inferred breakpoints.

Because mosaic analyses are frequently used in HIV-1 research, we also generated 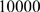 sequences by concatenating fragments from 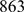 sequences of partial HIV-1 polymerase genes, spanning all of protease up to 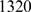 nucleotides of reverse transcriptase obtained from the Los Alamos HIV sequence database (http://hiv.lanl.gov). Each sequence was pre-screened using SCUEAL to ensure that only pure subtypes formed the base of this simulation. The number of fragments for each simulated sequence was drawn from a +1-shifted Poisson distribution with the mean of 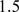 breakpoints/alignment; this guaranteed at least one breakpoint per alignment. The length of each fragment as a proportion of the total alignment length of 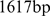 was determined using the stick-breaking process with beta distribution parameters 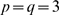. A 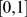 value was drawn from the beta distribution and the longest remaining fragment was split in that proportion to introduce each consecutive breakpoint into a sequence; if the shorter of the two resulting fragments was not at least 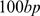 long, the proportion was rejected and the process was repeated with a new beta-distributed proportion. Simulated sequences were screened against an alignment of 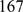 pure subtype reference sequences culled from our *pol* reference set (no CRFs were included in the reference). Note that because reference sequences were not identical to those used to generate the mosaics, this scenario simulated both recombination and mutational divergence found in HIV-1.

### A Surveillance Study

A bread-and-butter application of HIV subtyping algorithms is to characterize the subtype distribution in a cohort of patients or a geographic region and make inferences about the history and dynamics of HIV infection. We selected one of such recently published studies [48] that subtyped 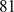 partial pol sequences from Bulgaria using REGA and found a diverse composition of subtypes, including three unassigned sequences.

### Database sequences

We downloaded all 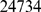 available reverse transcriptase sequences from the Stanford HIV drug resistance database, an ad hoc global sequence collection, that were (http://hivdb.stanford.edu/) annotated with one of the nine pure subtypes (or sub-subtypes e.g. A1), CRF01 (AE), CRF02 (AG) and applied SCUEAL to estimate what proportion of sequences may be unclassified inter-subtype recombinants, and the frequency of within-subtype recombination. The algorithm that currently performs database sequences annotation uses a neighbor joining phylogeny of the query sequence aligned to 100 reference sequences (spanning all group M subtypes and CRF01-CRF19) to assign the query sequence the subtype of the enclosing or nearest clade (R. Shafer, personal communication; also see [49]).

A total of 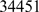 partial polymerase sequences from HIV infected individuals in the UK were available through the UK HIV Drug Resistance Database (www.hivrdb.org). This database is a central repository for HIV sequence data obtained in the course of routine clinical care and was established as a collaboration of 14 clinical centers and virology laboratories and 3 academic departments. The database acts as a resource for clinical, virological and epidemiological studies for the collaborating centres. The sequences released for analysis with SCUEAL had been fully anonymized and delinked and previously processed using REGA and Stanford [49] subtyping algorithms (Hughes GJ, Fearnhill E, Dunn D, et al. Molecular phylodynamics of the heterosexual HIV epidemic in the United Kingdom. PLoS Pathog. in process). We sought to compare the performance of SCUEAL to the other tools on a real-world task of automatic subtype classification of this complex sequence dataset assembled for population surveillance of a national HIV epidemic of significant subtype complexity.

### Implementation

The algorithms presented in this paper have been implemented as a collection of HyPhy [50] batch language scripts and can be dowloaded from http://www.hyphy.org/pubs/SCUEAL/. A README file explaining code usage and providing examples is included with the download. Simulated, biological and reference alignments and SCUEAL results can be downloaded from the same URL. An easy to use implementation of SCUEAL to screen up to 500 (this limit will be increased over time) sequences using a computer cluster maintained by the authors is available as a part of the Datamonkey http://www.datamonkey.org/ web server. Run times of SCUEAL on HIV-1 pol sequences depend on the complexity of the inferred mosaic type and take anywhere from 1–2 minutes for a pure subtype to up to an hour for a complex mosaic subtype on a desktop computer. Multiple query sequences can be screened in parallel if an MPI distributed environment is available. The screen of 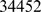 partial pol sequences from the UK drug resistance database took approximately 18 hours using 200 processors of an MPI cluster, translating to an average of 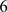 CPU/minutes per sequence.

## Results

### Simulation results

#### Parametric simulations

Parametric simulations tend to generate copious amounts of raw data (e.g. see Protocol S1) that are difficult to interpret directly, hence we generated a compact representation of simulation scenarios and results in Table 1 using a several descriptive metrics.

First and foremost one is interested how often is the correct mosaic (the order and identity of lineage assignments, e.g. 1-3-1-3-1 for the scenario in Figure 4) is recovered; this metric does not evaluate the accuracy of breakpoint placement. When an incorrect mosaic is reported, three types of classification errors are possible.

- A **subset** of the correct mosaic is recovered, i.e. some of the breakpoints are missed. For instance 1-3-1 would be a subset of the 1-3-1-3-1 mosaic. The method behaves conservatively in this case.
- A **superset** of the correct mosaic is recovered, i.e. in addition to all of the correct breakpoints spurious ones are inferred. For instance 1-3-1-3-1-1 would be a superset of the 1-3-1-3-1 mosaic. The method is overly liberal in this case.
- When the recovered mosaic is neither the subset nor the superset of the correct one, a **mismatch** has occurred. For example, 1-4-1-3-1 would be mismatched with 1-3-1-3-1. The method is inconsistent in this situation.

When a classification error occurs, it can either be because the GA failed to find the optimal solution, or because there is insufficient signal (due to small fragment length, low divergence etc) to infer the correct mosaic using the BIC criterion. The error due to the GA is an undesirable outcome, and we categorize each of the misclassified replicates into those which had worse fitness than the correct model (GA error) and those which had better fitness that the correct models (insufficient signal).

Second, we tabulated how often each of the correct breakpoints was recovered, and collected descriptive statistics about where the inferred locations were placed. A breakpoint was inferred ‘recovered’ if SCUEAL inferred at least one breakpoint, and the nearest inferred breakpoint to the simulated position involved correct parental lineages. For instance, a simulated A to B breakpoint at nucleotide 1000 would be counted as recovered in the inferred mosaic 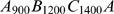, but not in 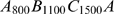.

The method has a very low rate of false positives correctly classifying 100/100 cases in Scenario 1 (no recombination). SCUEAL shows excellent operating characteristics when sequence divergence between parental strains and/or non-recombinant fragment length is sufficiently high; these two parameters approximate information content in the sequence. In scenarios 2,3,5 (except ancient recombination), 7 and notably, 11 (designed to simulate a typical HIV-1 CRF situation), SCUEAL assigned 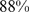 or more of replicates to the correct mosaic type; each of the breakpoints was also mapped very accurately with the standard deviation on the order of 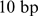. A very short non-recombinant fragment in scenario 4 (100 bp) made it difficult to detect recombination reliably; increasing the distance between parental strains dramatically increased the power, however from 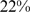 for close parents to 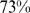 for distant parents.

Ancestral recombination involving interior branches in the tree (e.g. see Protocol S1) also complicated mosaic classification because of weaker phylogenetic signal. In all three scenarios with the ancient option (2,3 and 5), the proportion of correctly identified mosaics was lower than for extant parental lineage situation, but in most missed (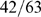) cases the assigned mosaic had a better BIC score - suggesting lack of phylogenetic signal as the main source of error. Overall, the ability of SCUEAL to accurately describe over 50% of mosaics due to ancient recombination is encouraging as many HIV-1 CRFs appear to be the result of ancient recombination, i.e. they fail to unambiguously cluster with any of the reference “pure” subtypes.

The complex pattern in scenario 6, where 10 non-recombinant fragments of length 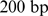 each, made concurrent detection of all 9 breakpoints difficult (30% for close parents and 64% for divergent strains). However, this was mostly due to one or two missed breakpoints–the average accuracy of mapping each individual breakpoint was high (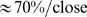, 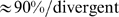), with no single breakpoint (e.g. in the middle of the alignment vs close to one of the ends) missed at an abnormally high frequency. Scenario 6/close, is the only scenario (many short fragments with relatively close parental strains) where the majority 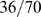 of classification errors were due to premature GA termination; this could be improved by adjusting GA parameters at the expense of longer run times.

SCUEAL could not detect recombination in sequences with very low (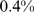) parental strain divergence (scenarios 8/close and 9/close), and had low (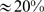) power in breakpoint detection for 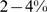 divergent strains in scenarios 8/divergent, 9/divergent and 10, overwhelmingly due to lack of phylogenetic signal and not to premature GA convergence.

Each individual detected breakpoint was on average very close (standard deviations in the range of 5–20 bp) to a true breakpoint, confirming that SCUEAL produces a high-resolution breakpoint map.

#### HIV pol simulations

Using the classification defined in the previous section, SCUEAL performance on 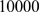 simulated data sets can be summarized thus: 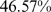 correct sequence mosaics (i.e. each breakpoint and correct lineage) were recovered, 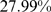 recovered mosaics were supersets (extra breakpoints) of the correct type, 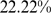 - subsets (missed breakpoints) of the correct type, and 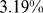 - mismatched. Overall, 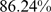 of the replicates were correctly identified as recombinant strains. Of 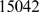 simulated breakpoints, 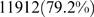 were recovered correctly, with a median distance between the simulated and the inferred breakpoint of 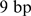 (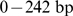 for the 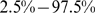 range). Median level of model averaged support for the inferred mosaic was estimated at 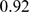.

**Figure 5:**
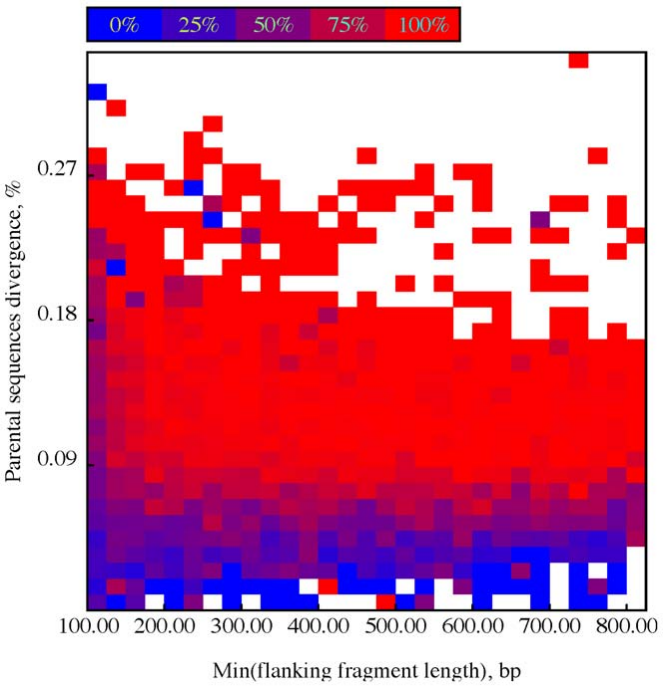
Power and accuracy in the sequence shuffling simulation. Power of SCUEAL to detect breakpoints in the HIV-1 pol sequence shuffling scenario as a function of recombinant fragment length (x-axis) and divergence between parental strains (y-axis). Grid cells are colored according to the proportion of correctly detected breakpoints (different cells may summarize different numbers of simulations). White squares are plotted when there were no simulated breakpoints within a corresponding length-divergence range of values.

However, these numbers alone do not present the complete picture of how the method performed - the power to detect recombination is significantly dependant upon the length of recombinant strains (e.g. a 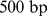 fragment is easier to detect than a 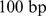 one on average), and the relative level of divergence between parental strains (e.g. inter-subtype recombination is easier to detect than within-subtype). To capture these dependancies, we binned all breakpoints in simulated strains by the length of the shorter of the flanking fragments and the pairwise genetic distances between parental strains over that fragment. For example the breakpoint in a mosaic of type A–B with the 500 bp coming from subtype A and 200 bp coming from subtype B, would contribute to the bin with 200 bp length and the genetic distance between strain A and strain B over the last 200 bp of the sequences. We next plotted detection power, i.e. the proportion of times a correct-type breakpoint (e.g. A–B for the previous example) was inferred within 100 bp of the simulated breakpoint (see Fig. 5). The power of the method to detect a breakpoint grows with the length of the flanking recombinant fragments and the genetic distance of the two parental strains. For example, 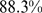 of all breakpoints generated from sequences more than 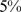 divergent and involving fragments of at least 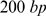 on either side were correctly identified. The fraction increased to 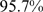 for 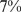 or greater divergence and at least 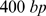-long fragments–values encountered with commonly annotated inter-subtype recombinant mosaics in HIV.

### Surveillance study

**Figure 6:**
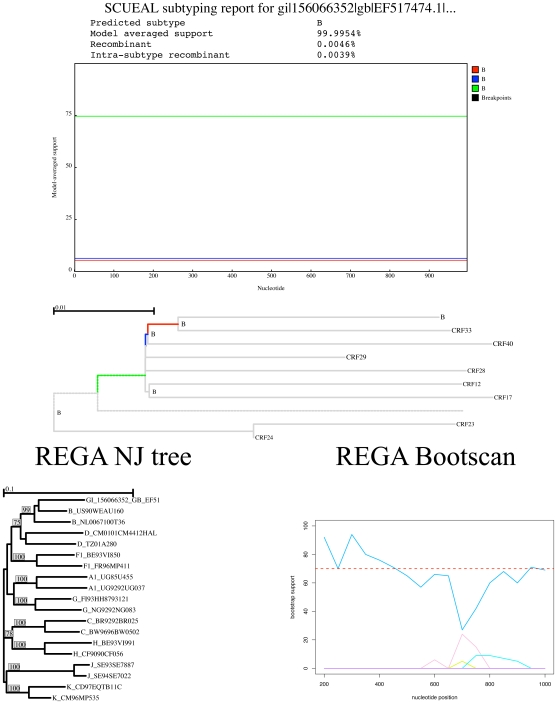
An example of a good agreement between SCUEAL and REGA in classifying a partial pol subtype B sequence. The SCUEAL clustering plots present in this figure and Figures 7, 8 and 9 are conceptually analogous to bootscan plots, i.e. which reference sequence is the most likely sister lineage of the query sequence for a given site, but is based on model averaged support values instead of phylogenetic bootstrap. A partial reference tree with placed query is shown; color coding is consistent between the similarity plot and the tree. A phylogenetic tree with bootstrap support values and bootscan plot using the REGA alignment generated for the query sequence are shown.

The results of SCUEAL and REGA screening of 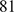 partial polymerase sequences isolated from patients in Bulgaria [48] were quite similar, yet revealingly different in some cases. The methods concurred on 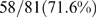 sequences, reporting 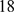 subtype A sequences, 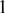
–subtype B, 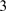
–CRF01 (AE), 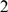 each of C and G, and one of subtype 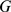 and 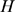. Figure 6 depicts a query sequence on which the methods agreed well. Both the neighbor joining tree and the bootscan plot based on the automatic alignment produced by REGA indicate strong clustering with the B clade and lack of evidence for recombination, yielding an assignment confidence of 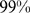. Concordantly, SCUEAL reports a 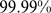 model averaged support for clustering with a clade B sequence, although there is a bit of uncertainty which exact lineage the query should be grafted on.

There are several kinds of disagreement between REGA and SCUEAL classification results.

#### Unassigned sequences

**Figure 7:**
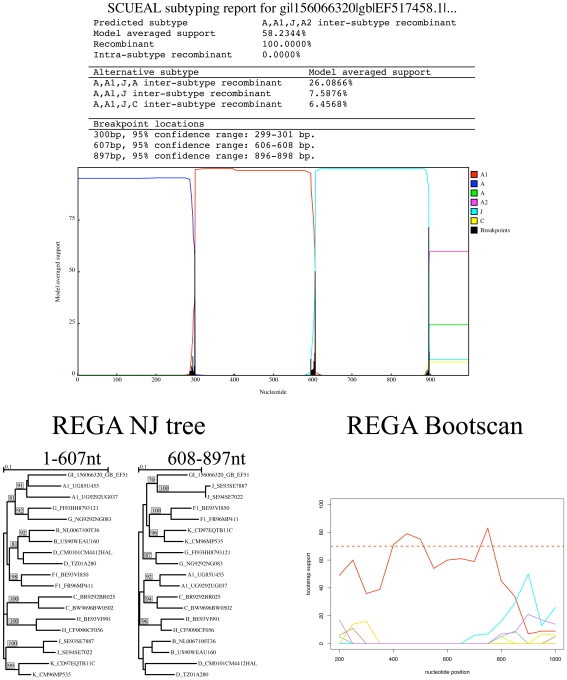
An instance when a sequence unclassified by REGA is inferred to be a novel recombinant form by SCUEAL; the A–J mosaic structure is also confirmed by trees and bootscan plots based on the REGA reference alignment.

REGA did not assign a subtype to six sequences in the sample. This happens either when there is insufficient phylogenetic bootstrap support for clustering with a pure subtype or CRF reference, or when bootscan detects a recombinant form that is not well explained by an existing CRF. Because SCUEAL uses a much larger reference alignment than REGA (e.g. there are 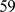 sequences in the greater B clade, including a number of CRF fragments that cover parts of the pol gene, vs 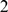 in the default REGA) alignment, it was able to assign 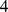 of the 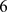 sequences to subtype 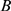 with high (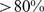) confidence. Interestingly, these sequences were grafted onto interior branches of the 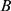 clade, highlighting the intrinsic power of SCUEAL of being able to make full use of the fixed reference topology. The remaining two sequences were classified as novel recombinant forms, in congruence with the bootscan profile. For example, in Figure 7, a novel A–J recombinant is reported by both methods, but REGA's conservative assignment scheme would still report this case as unassigned. SCUEAL proposes several A–J type recombinant forms, with A-A1-J-A2 being the best supported one; overall there is 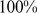 model-averaged support for presence of recombination in this sequence. Due to a much larger set of subtype A reference sequences, our approach is capable of a more precise characterization of the mosaic, whose breakpoints are mapped very accurately (to 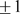 base pair). The sliding window nature of phylogenetic bootscanning (REGA uses a 400 bp window with a 50 bp stride by default) does not naturally permit precise breakpoint mapping. Splitting the sequence along the A–J boundary and building traditional neighbor joining trees using the REGA reference alignment, confirms the structure predicted by SCUEAL.

#### Within-subtype recombination

**Figure 8:**
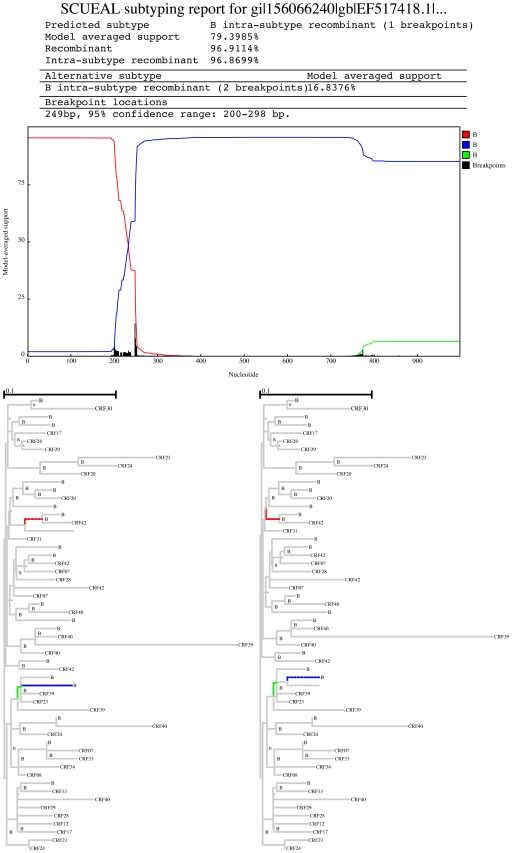
An example of within-subtype (B) recombination detected by SCUEAL, but not by REGA. A partial reference tree with placed query is shown; color coding is consistent between the similarity plot and the tree.

Seven of the discordant results occurred when a sequence classified as pure subtype by REGA was identified as within-subtype recombinant by SCUEAL. An example of this is shown in Figure 8, where the putative parental strains are approximately 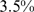 divergent on the tree.

#### Missed recombinants

**Figure 9:**
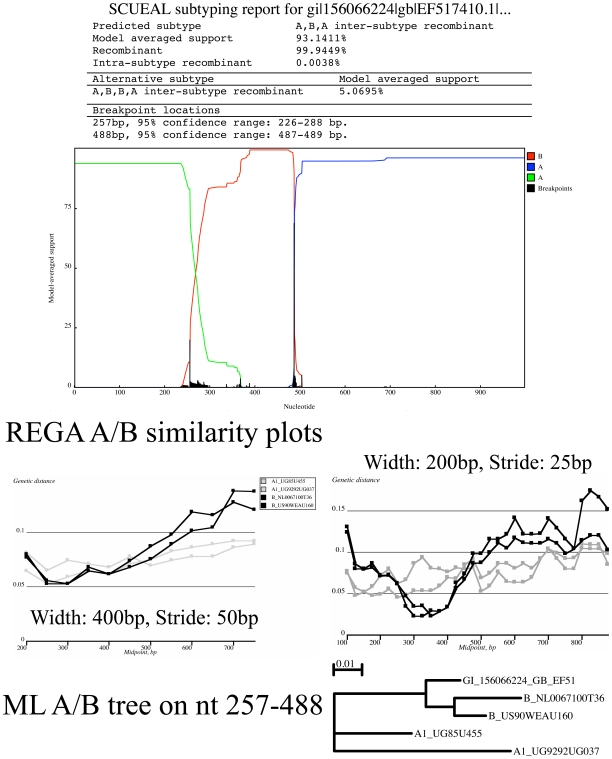
An instance when a sequence assigned to subtype A by REGA is deduced to be an A-B-A mosaic by SCUEAL. Similarity plots based on the reduced REGA alignments (only A and B subtype reference sequences) confirm that the same mosaic structure is supported using if a small enough window is selected for a sliding window analysis.

The remaining 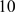 mismatches arose when a pure subtype sequence (according to REGA) was instead reported as an inter-subtype recombinants with very strong (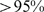) model-averaged support for recombination. The obvious explanation for why REGA may be missing these recombinants is that the size of the sliding window used for bootscanning (400 bp) limits how short individual mosaic fragments can be. This limitation becomes relevant for single gene recombination analysis, when the total length of the sequence is on the order of 500–1000 bp. The A-B-A mosaic example in Figure 9 was classified as subtype A by REGA. However, adjusting the sliding window parameters from to use window size of 200 bp instead of 400 bp and stride 25 bp instead of 50 bp revealed that subtype B sequences from the REGA reference alignment were genetically closer to the query than subtype A sequences over the segment predicted by SCUEAL to cluster with subtype B. Furthermore, a maximum likelihood tree (exhaustive search) on that segment supports the same clustering.

### Stanford database sequences

SCUEAL analyses indicate that while a majority of sequences annotated as pure subtype in the Stanford drug resistance database are assigned to a correct subtype, a substantial proportion (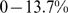 depending on subtype) are better explained as circulating or unique recombinant forms (CRF/URF) and a similar proportion appear to be within-subtype recombinants (Table 2). Importantly, there are only a few cases when SCUEAL infers a pure subtype sequence which is annotated with a different pure subtype in the database. For instance, out of 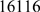 subtype B sequences there were 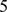 subtype D sequences, two–subtype J and two–subtype A, hence the vast majority of potentially misclassified subtypes in the database are due to recently characterized CRFs and URFs which are partially derived from the database subtype. When SCUEAL infers recombination, model averaged support for at least one breakpoint is very strong (median 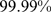, mean 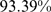, 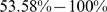 for the 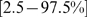 range), but the inference of the exact mosaic type is less certain on average (median 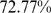, mean 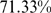, 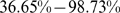 for the 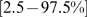 range), which is not surprising given that many of the sequences are quite short.

**Table 2:** SCUEAL screening results on partial HIV-1 reverse transcriptase sequences from the Stanford Drug Resistance database.

Subtype	Sequences	Agree	within-subtype	Diff. pure subtype	Diff. recombinant	Top 3 CRFs and URFs
A	1740	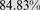	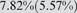	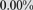	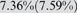	CRF33/34 (31); A1,D (14); AE, B (7)
B	16116	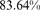	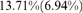	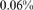	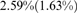	CRF28/29 (273); CRF42 (54); CRF20/23/24 (30)
C	3133	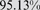	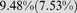	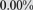	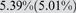	B,C,CRF31 (56); B,C (36); C/CRF07 (8)
D	624	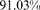	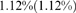	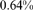	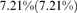	A1,D (16); B, CRF19 (4); B, D (3)
F	464	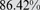	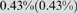	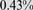	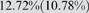	B,F1 (27); CRF29, F1 (5); B, CRF40, F1 (4)
G	757	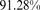	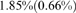	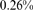	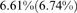	B, CRF14 (4); B, G(3); G,J (3)
H	28	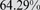	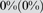	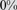	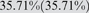	G,H (2); A, H (1); A, B, K (1)
J	22	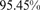	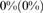	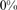	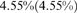	C,J (1)
K	166	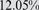	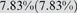	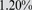	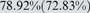	CRF32, G (22); CRF30, CRF32 (7); C, CRF32 (5)
CRF01 (AE)	1552	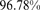	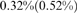	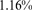	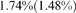	CRF22 (5), AE,B (4); B, CRF33 (4)
CRF02 (AG)	1352	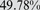	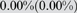	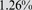	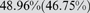	A,G (285), A,CRF36,G (41), A,CRF02,G (34)

**Subtype** lists the sequence subtype as annotated in the database. **Sequences** provides the number of sequences downloaded from the database. **Agree** gives the percentage of sequences for which SCUEAL returned the same subtype as that stored in the database. **within-subtype**–SCUEAL inferred within-subtype recombination within the same subtype as the one stored in the database; figures in parentheses show the proportion of within-subtype recombinants identified when DRAM positions were masked. **Diff. pure subtype**–the proportion of cases where SCUEAL inferred a pure subtype different from the annotated one. **Diff. recombinant**–the proportion of cases where SCUEAL inferred a recombinant mosaic with at least one fragment different from the annotated subtype; figures in parentheses show the proportion of within-subtype recombinants identified when DRAM positions were masked. **Top 3 CRFs and URFs**–three most frequent mosaics inferred by SCUEAL.

Agreement for subtypes H and K is unusually poor, however there are only a few sequences assigned to this subtype, and a small number of existing reference samples to base inference upon. In particular, many sequences annotated as subtype K appear to have been partly derived from CRF30 and CRF32 strains. Over 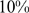 of sequences annotated as subtype F are classed as B,F (or partial CRFs) recombinants by SCUEAL, but this can be expected as there are at least seven known CRFs (17, 28, 29, 38–40, 42) that are comprised of B and F mosaics with one or more breakpoints in the pol gene. For CRF02-annotated samples, 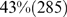 of the sequences that were classified differently by SCUEAL as A,G recombinants appear to support breakpoints that are different from those included in the reference CRF02 strains. This could indicate that a larger sample of CRF02-like reference strains may be necessary to accurately capture the diversity of these viral strains.

HIV evolution in the era of Highly Active Antiretroviral Therapy (HAART), especially in the developed world, is significantly influenced by selective forces that favor viral strains with mutations that confer drug resistance in the presence of a corresponding drug. This is especially true of subtype B viruses, circulating in North America and Western Europe, where HAART has been exerting well-characterized selective pressure on the virus for over a decade [51], leading to increasing prevalence of HIV strains that harbor drug resistant associated mutations (DRAM, e.g. [52],[53]). Convergent evolution to acquire DRAM can have a confounding effect on phylogenetic subtyping methods, by making regions rich in DRAM appear closely related in evolutionarily distant strains and potentially leading to a false signal of within- (or inter-) subtype recombination. To assess this effect, we identified subtype B RT sequences (as annotated in the database) that harbored at least one known DRAM [51] (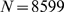) and reran SCUEAL on these sequences after replacing all DRAM with missing data (3 in-frame gaps for each DRAM codon, e.g. any codon at position 215 in reverse transcriptase that encodes an 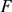 or a 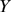). Between 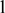 and 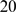 positions (median 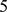) per sequence were masked by this procedure. DRAM masking substantially reduced the number of sequences that were classified as within-subtype recombinants, taking the number down from 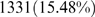 to 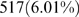. For other subtypes, where the frequency is of DRAMs is lower than in subtype B sequences, the effect of masking DRAMs on the proportion of inferred intra-subtype recombinants (and other recombinant forms) is much more muted (Table 2). Consequently, convergent evolution to acquire drug resistant mutations appears to be a significant factor contributing to the within-subtype recombination signal, although the reduction in phylogenetic signal due to fewer informative sites in masked sequences is also a possible cause of this effect.

### Large scale subtype classification in a surveillance and epidemiological linkage study

The comparison between SCUEAL and REGA on this data set (see Table 3), is similar to what was observed for the Stanford dataset. For well sampled subtypes (A,B,C,D,F,G,AE) the agreement between the methods was good to excellent (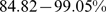), with a noticeable proportion (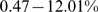) of within-subtype recombinants. Note that the proportion of within-subtype recombinants was not as significantly affected by masking out DRAMs as discussed in the previous section; for example the proportion was reduced from 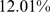 to 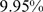 for subtype B sequences, and actually increased for subtype 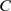 sequences. This could be because the UK sequences are longer than (both protease and reverse transcriptase) than the Stanford sample (reverse transcriptase only). Also, because SCUEAL is a stochastic algorithm, some variation (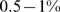 in our simulation experiments, results not shown) between runs due to the indeterministic nature of the algorithm, especially between “borderline” sequences (those sequences that have a weak support for a the inferred mosaic), is to be expected. Small proportions of inter-subtype recombinants called by SCUEAL were not not identified by REGA. For CRF02 and CRF06, the agreement was quite poor, however the discord is easy to explain. For CRF02 SCUEAL identified many A,G recombinants but with breakpoints differing from those mapped for CRF02 (note that it is likely that G is the recombinant strain, but we refer to CRF02 as the recombinant to maintain compatibility with the current nomenclature); other CRF strains that include CRF02 - like fragments in pol (CRF30, CRF36) account for most of the other discrepancies. For sequences typed as CRF06 by REGA, the majority of SCUEAL classification involve CRFs derived from CRF06 (e.g. CRF30, CRF32).

**Table 3:** SCUEAL screening results on partial HIV-1 polymerase sequences from the UK.

Subtype	Sequences	Agree	within-subtype	Diff. pure subtype	Diff. recombinant	Top 3 CRFs and URFs
A	2119	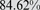	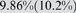	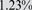	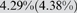	CRF22 (24); A1, D (12); A1, C (4)
B	19871	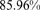	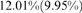	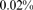	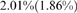	B, D (120); B, CRF03 (40); B, F1 (38)
C	7381	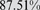	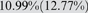	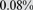	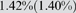	B, C (11); C, D (11); C, J (10)
D	614	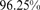	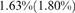	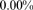	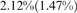	B, D (3); D, K (2); A, D (2)
F	110	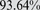	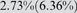	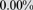	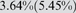	B,F (2); F, G (1); F, H (1)
G	673	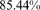	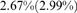	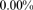	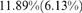	F1, G (25); CRF30, G (10); A, G (10)
H	35	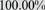	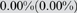	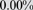	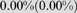	
J	35	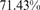	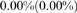	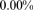	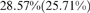	B, J (3); CRF09, J (3); G, J (2)
CRF01 (AE)	419	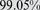	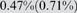	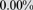	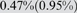	AE, B (2)
CRF02 (AG)	1014	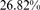	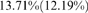	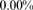	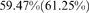	A, G (278); A, CRF30, G (72); A, CRF30, CRF36 (56)
CRF06	147	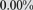	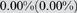	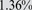	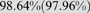	CRF32, K (34); CRF32, G (23); CRF30, CRF32 (14)

**Subtype** lists the sequence subtype as annotated in the database. **Sequences** provides the number of sequences downloaded from the database. **Agree** gives the percentage of sequences for which SCUEAL returned the same subtype as the one inferred by REGA. **within-subtype**–SCUEAL inferred within-subtype recombination within the same subtype as the one inferred by REGA; figures in parentheses show the proportion of within-subtype recombinants identified when DRAM positions were masked. **Diff. pure subtype**–the proportion of cases where SCUEAL inferred a pure subtype different from the REGA assignment. **Diff. recombinant**–the proportion of cases where SCUEAL inferred a recombinant mosaic with at least one fragment different from the annotated subtype; figures in parentheses show the proportion of within-subtype recombinants identified when DRAM positions were masked. **Top 3 CRFs and URFs**–three most frequent mosaics inferred by SCUEAL.

Of 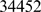 sequences, a non-trivial proportion 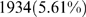 were not classified by REGA, with 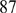 of those also not classified by the HIVdb subtyping algorithm. According to SCUEAL 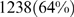 were URFs, and the remainder–pure subtypes of CRFs. Among 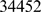 sequences, SCUEAL identified 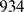 complex recombinant forms (more than 3 constituent sub- or subsubtypes) and 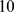 URFs with at least 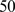 sequences each, including:

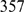
 G,A and 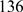 G,CRF02 recombinants. Given the degree of uncertainty about mapping the breakpoints in CRF02 (the nomenclature here is confusing, because recent evidence suggests that G was derived as a recombinant of A and CRF02 sequences) reference sequences, these sequences can be thought of as a A,G, CRF02 recombinants sequences. The finding also indicates that the diversity of this clade is quite significant, and reference data sets may need to be enriched for A, G and CRF02 sequences to enable more accurate subtype assignment. REGA assigned 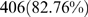 of these sequences to subtype CRF02, 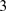 to subtype A(A1), 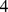 to G, one to subtype 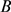 and did not classify 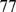 sequences.

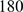
 A1,D recombinants. 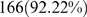 of those were not definitively classified by REGA, with the remainder assigned to A1 (12), D (1) and CRF10_CD (1). There are several precedents for this type of mosaic structure, including CRF16, CRF19 and CRF35 which all have a mosaic AD structure in pol.

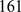
 B,CRF39 recombinants. Because most of pol sequence in CRF39, which is found circulating in Brazil, is mapped to subtype B [54], this form can be reported as a B intra-subtype recombinant. This finding also illustrates the capacity of SCUEAL to map within-subtype diversity with high resolution. Almost all (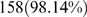) of those are identified as subtype B sequences by REGA, which is correct if within-subtype recombination is discounted.

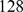
 B,D recombinants, 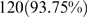 of which were classified as subtype B by REGA. Because B and D subtypes are closely related (compared to other between-subtypes comparisons), this mosaic type is difficult to detect.

## Discussion

We present a new phylogenetic method (SCUEAL) to automatically determine a subtype and map the recombinant structure in HIV-1 sequences. Our method uses a statistically robust maximum likelihood multi-model inference approach to examine tens of thousands of potential mosaic structures in a single run guided by an evolutionary algorithm, identify those well supported by the data and quantify the reliability of all estimated quantities. SCUEAL is designed to handle the inclusion of recombinant strains in reference alignments, operate on large reference alignments with minimal loss of speed and permit easy expansion of existing reference alignments as new subtypes or circulating recombinant forms.

Using an extensive collection of simulated sequence alignments, covering a wide range of evolutionary parameters and including biological HIV-1 sequences, we determined that the method was capable of accurate detection of the number and location of recombination breakpoints as well as appropriate parental lineages, given sufficient sequence divergence. For non-parametrically generated HIV-1 pol mosaics, the recovery rate of breakpoints was 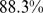 for 5% or greater divergence between parental strains and 200 bp or longer sequence fragments. On average, individual breakpoints were inferred within 10 bp of the simulated locations. SCUEAL had a 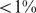 rate of false positives on parametrically simulated data.

A comparison with a popular phylogeny based rapid subtyping tool REGA [18] on an HIV-1 pol surveillance dataset [48] illustrated that SCUEAL was able to automatically detect recombinant sequences with short mosaic fragments, classify and map unknown mosaic types and resolve cases that confounded REGA. A large scale screen of 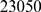 database sequences revealed that approximately 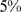 of pure subtype reverse transcriptase sequences show evidence of within-subtype recombination and a further 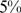 are likely novel or known circulating recombinant forms, highlighting the need for more precise determination of subtype information for public databases. Because up to 10% of HIV-1 infections occur with Unique Recombination Forms (URFs) when superinfection with divergent strains is relatively common (e.g. [55],[56]), the ability of SCUEAL to automatically annotate such forms is of critical importance. Furthermore, many evolutionary analyses, such as dating and selection screens, can be biased by the inclusion of recombinant sequences without necessary corrections [57],[58]. Studies that seek to identify clinical and evolutionary differences between different HIV subtypes (e.g. [59],[60]) also rely on the accurate classification of subtypes for all input sequences. To our knowledge, none of the existing subtype classifiers are designed to detect within-subtype recombination, which is in all likelihood much more frequent than inter-subtype recombination because sufficiently divergent strains of the same subtype routinely co-circulate in host populations (e.g. [61]) and within-host sequences often present phylogenetic evidence of extensive recombination [62]. We note that convergent evolution to acquire drug resistance associated mutations appears to have a strong confounding effect on detecting within-subtype recombination and should be accounted for if the focus of the analysis is to identify within-subtype recombination in regions of HIV that include many such mutations.

SCUEAL provides an automatically determined mosaic structure for any input sequence, including the cases when existing methods fail to derive such a structure. While this feature is a qualitative advance over existing approaches, it may also invite over-interpretation of computational results, and we emphasize that this should be avoided. Consider for example, the strain presented in Figure 7. SCUEAL results allow us to deduce that the strain is an inter-subtype recombinant with a high degree of confidence (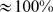). The analysis also strongly implies that 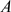 and 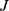 strains or their ancestors contributed segments of the pol gene to the query sequencer, but also reports several credible mosaic forms that could be assigned to the strain, counter-indicating a definitive (e.g. A-A1-J-A2) mosaic determination. We would like to stress that SCUEAL determination of a novel recombinant form should not lead the users to automatically declare the sequence as such, but rather as an invitation to perform further examination of the data, perhaps with a specialized reference alignment, enriched for the subtypes detected by SCUEAL. Continuing with the example, the combination of A and J subtypes in one sequence is not uncommon (e.g. CRF06, CRF11, CRF13, CRF27) and extensive mosaicism in the pol gene has also been reported previously [63]. Moreover, the “J” clade in the SCUEAL reference alignment also contains J-like segments from several CRFs that circulate more widely that pure subtype J strains confined primarily to Central and West Africa [4]. Whether or not the segment assigned to clade J may instead belong to an unsampled clade of HIV-1 cannot ultimately be determined with the currently available estimate of HIV-1 diversity. Subtype classification is extensively used as a tool in molecular epidemiology and in surveillance studies of HIV because of their association with different populations. Multiple subtypes were detected in the UK in 1995 [64] and by 2007, non-B subtypes comprised the majority of new diagnoses in the UK [65],[66]. In addition, subtype classification is important for clinical reasons in HIV because of biological differences that have been observed with respect to rate of progression to disease [67], and patterns of drug resistance mutations [68],[69]. For that reason, sequences in the UK HIV Drug Resistance Database are routinely subtyped before analysis. The rapid increase in scale of the task (the current database release contains over 50,000 sequences) and the range of diversity of the subtypes and recombinants now present in the UK epidemic highlights an urgent need for an automated, informative, reliable and rapid method for classification on the sequence data collected that will scale to hundreds of thousands of sequences on commodity distributed computing platforms.

Empirical datasets in this study were limited to the partial polymerase gene of HIV, partly because this genetic region routinely sequenced for surveillance and diagnostic purposes, has few easily aligned indels–thus avoiding potential biases due to unreliable automatic multiple sequence alignment (e.g. [46]), and contains many of the breakpoints mapped for known CRFs. However, SCUEAL can use any reference alignment, including full length HIV-1, Hepatitis C virus, Influenza A virus genomes and non-viral sequences, and we plan to implement this functionality in future versions of SCUEAL.

Finally, we would be remiss to overlook some of the limitations of our approach. SCUEAL is a fairly computationally demanding method, and consequently is considerably slower that some other screening tools. Parallel execution on a computer cluster can mitigate this issue and permit one to process thousands of sequences per hour. As any method that is based on a reference alignment, SCUEAL is susceptible to biased inference if the reference alignment is inaccurate or if reference sequences are themselves misclassified. We took a number of precautions to ensure that the reference alignment was accurate by focusing on an easily alignable genomic region, a conservative automatic alignment procedure for the query sequence and an incremental algorithm for adding and accurately labeling reference sequences. SCUEAL uses a nucleotide evolutionary model to fit phylogenetic likelihood models for the sake of computational efficiency and this could lead to difficult to quantify biases in mosaic structure mapping; more realistic models (e.g. codon models) can be “plugged-in” without any alteration to the methodological framework if desired.

## Supporting Information

Protocol S1 Settings and results for each of the parametric simulation scenarios
(4.08 MB PDF)
