# Supplementary material for: An Evolutionary Model-Based Algorithm for Accurate Phylogenetic Breakpoint Mapping and Subtype Prediction in HIV-1
Source: PLoS Comput Biol. 2009 Nov 26;5(11):e1000581. doi: 10.1371/journal.pcbi.1000581 (PMC2776870; doi:10.1371/journal.pcbi.1000581)
Supplement: Protocol S1 — Settings and results for each of the parametric simulation scenarios (4.08 MB PDF) [file pcbi.1000581.s001.pdf]

# 1 Parametric simulation scenarios

The top panel of each figure contains a graphical summary of SCUEAL results averaged over 100 replicates. The abscissa is labeled with sequence coordinates and the ordinate - with doubly averaged (Akaike weights model-aveage for each replicate and then arithmetic average over all replicates) support of having the query sequence cluster with a particular lineage in the tree at a given position in the alignment. Ancestral lineages are annotated by a slash separated list of all the daughter lineages (e.g. 7/8 is the parental lineage of 7 and 8) The dot plot indicates double averaged support for placing a breakpoint at a given position in the alignment.

There are also two tables in each plot. The first one is showing how many replicates were classified correctly, were a sub/superset of the correct mosaic, or were assigned a mismatched mosaic. For each category, the 'Better or Equal BIC/Delta(BIC)' column includes the number of replicates whose assigned mosaic had a better BIC than the generating mosaic, and the median (2.5%-97.5% range) of difference in BIC between the inferred and the correct mosaics. The second one tabulates how often each of the simulated breakpoints was found, and where it was placed by SCUEAL

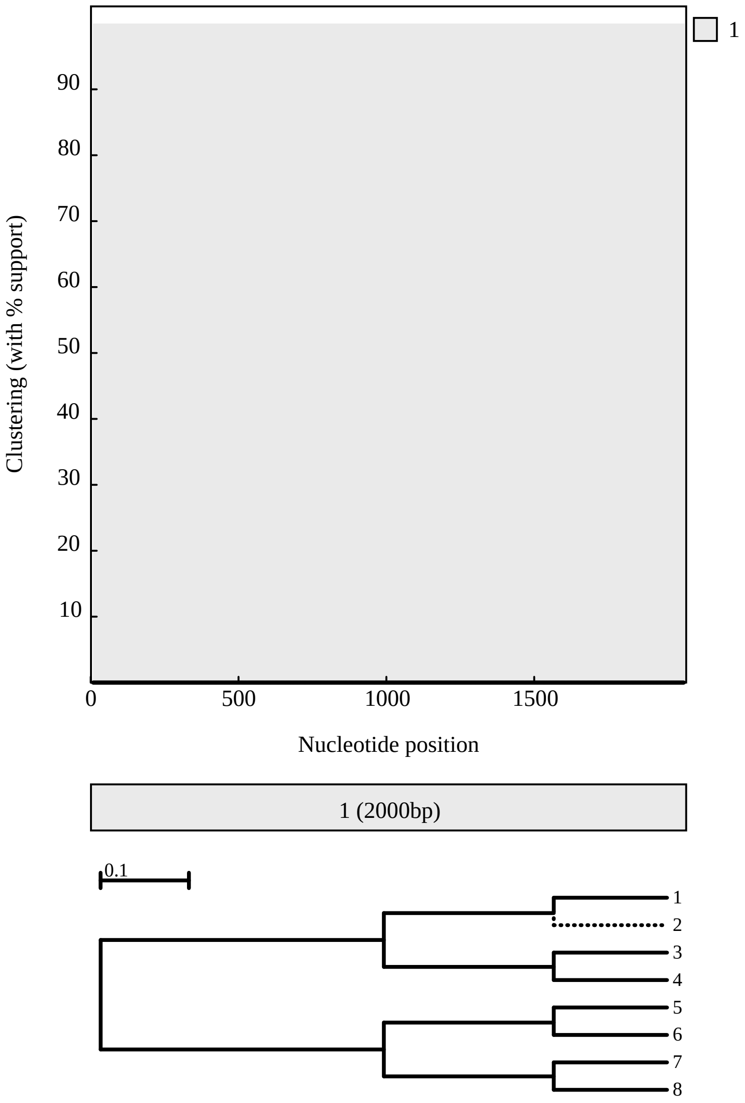


| Classification | Count | Better or Equal BIC/Delta(BIC) |
|---|---|---|
| Correct | 100 | 100/22.80 (22.80,22.80) |
| Subset | 0 | |
| Superset | 0 | |
| Mismatch | 0 | |

Figure S1: Scenario 1. No recombination

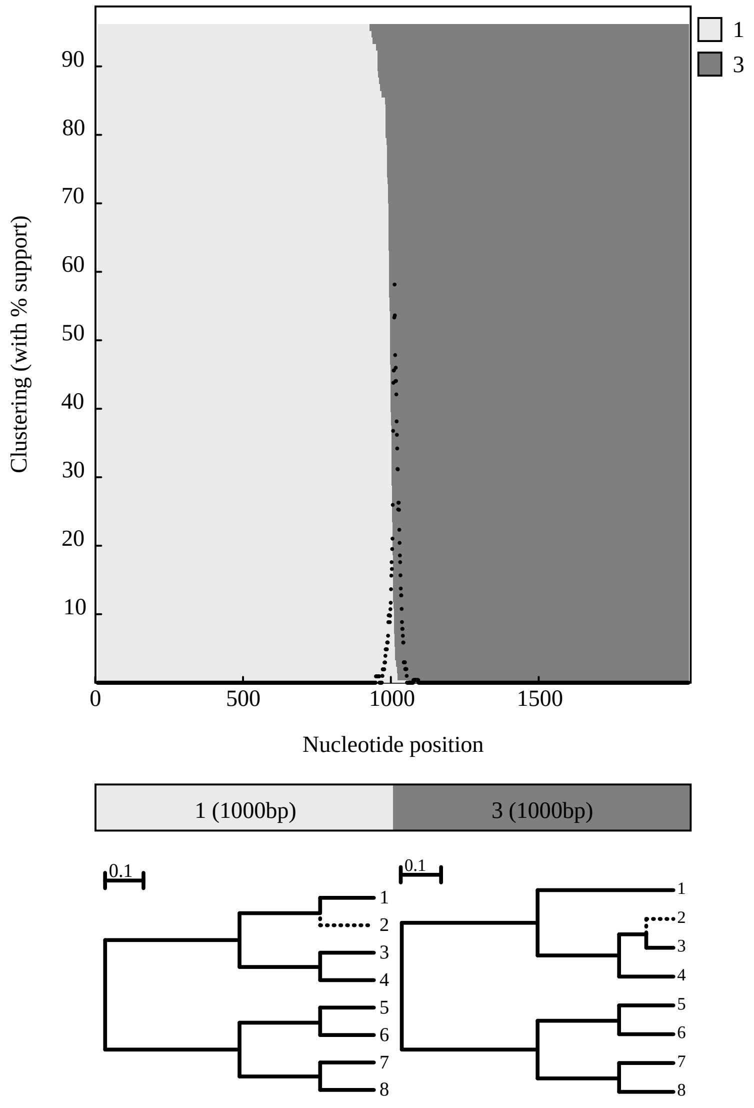


| Classification | Count | Better or Equal BIC/Delta(BIC) |
|---|---|---|
| Correct | 100 | 88/ 0.80 (-0.44, 7.78) |
| Subset | 0 | |
| Superset | 0 | |
| Mismatch | 0 | |

| Breakpoint | Times found/ Location (median, st.dev (2.5%-97.5%)) |
|---|---|
| 1000bp 1:3 | 100/990,18.99 (931,1015) |

Figure S2: Scenario 2. An evident breakpoint/close

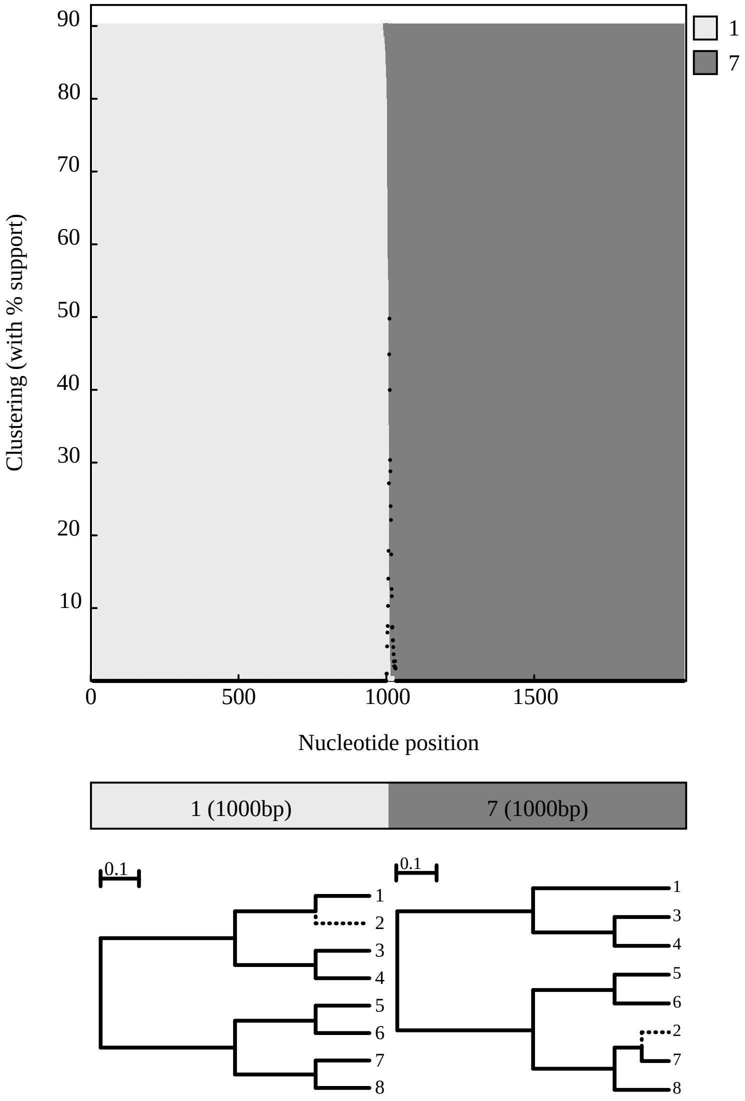


| Classification | Count | Better or Equal BIC/Delta(BIC) |
|---|---|---|
| Correct | 100 | 75/ 0.00 (-0.33, 8.35) |
| Subset | 0 | |
| Superset | 0 | |
| Mismatch | 0 | |

| Breakpoint | Times found/ Location (median, st.dev (2.5%-97.5%)) |
|---|---|
| 1000bp 1:7 | 100/1000, 5.04 (987,1007) |

Figure S3: Scenario 2. An evident breakpoint/divergent

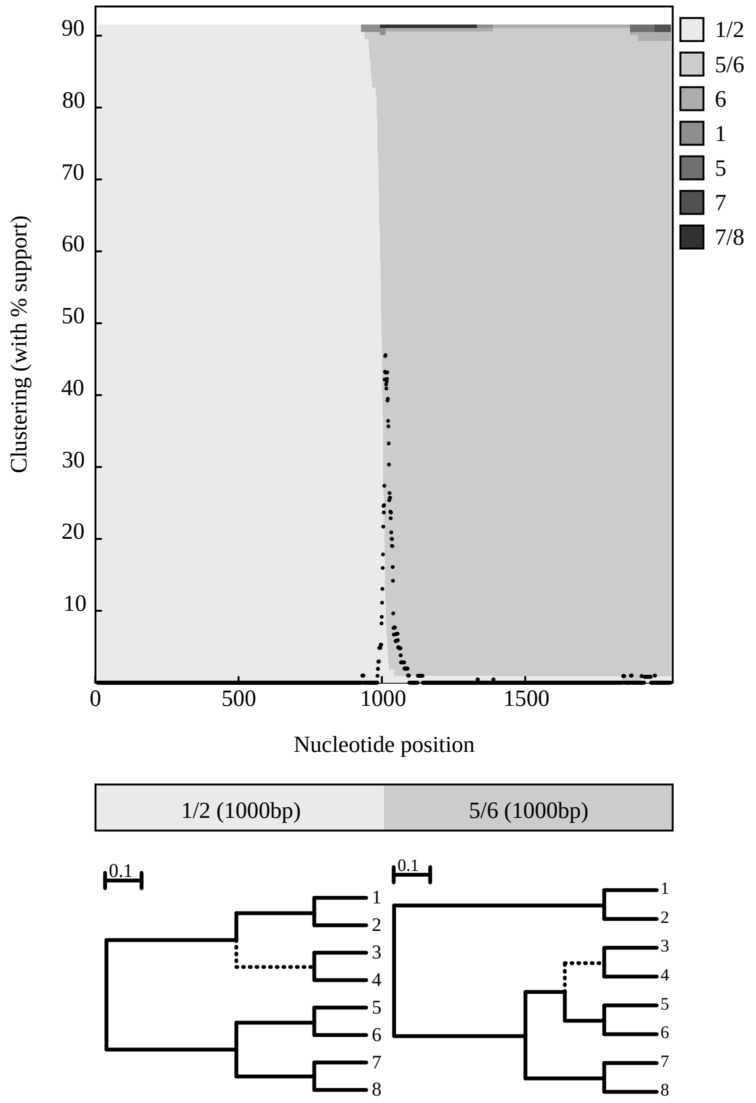


| Classification | Count | Better or Equal BIC/Delta(BIC) |
|---|---|---|
| Correct | 92 | 86/ 1.69 (-0.04, 8.82) |
| Subset | 0 | |
| Superset | 7 | 6/ 4.53 (-6.59,12.13) |
| Mismatch | 1 | 1/ 1.48 ( 1.48, 1.48) |

| Breakpoint | Times found/ Location (median, st.dev (2.5%-97.5%)) |
|---|---|
| 1000bp 1/2:5/6 | 96/992,16.62 (947,1017) |

Figure S4: Scenario 2. An evident breakpoint/ancient

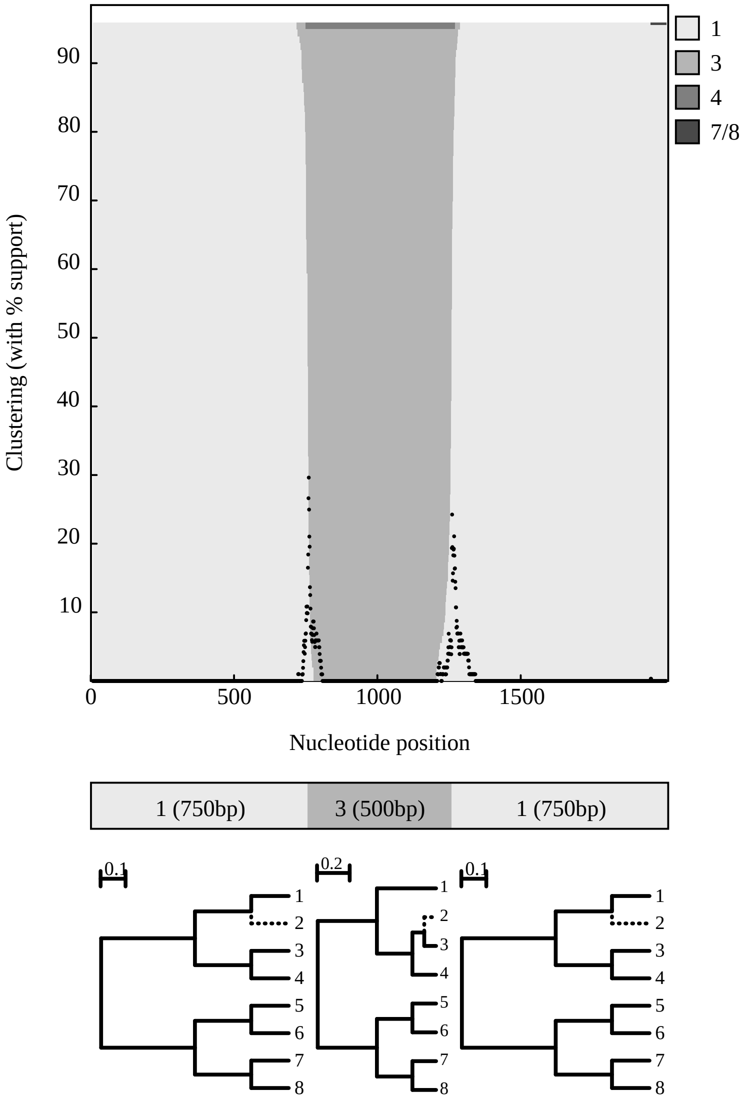


| Classification | Count | Better or Equal BIC/Delta(BIC) |
|---|---|---|
| Correct | 98 | 96/ 4.00 ( 0.00,14.03) |
| Subset | 0 | |
| Superset | 1 | 1/ 4.49 ( 4.49, 4.49) |
| Mismatch | 1 | 1/10.34 (10.34,10.34) |

| Breakpoint | Times found/ Location (median, st.dev (2.5%-97.5%)) |
|---|---|
| 750bp 1:3 | 99/749,10.23 (720,769) |
| 1250bp 3:1 | 99/1251,15.26 (1201,1273) |

Figure S5: Scenario 3. Two evident breakpoints/close

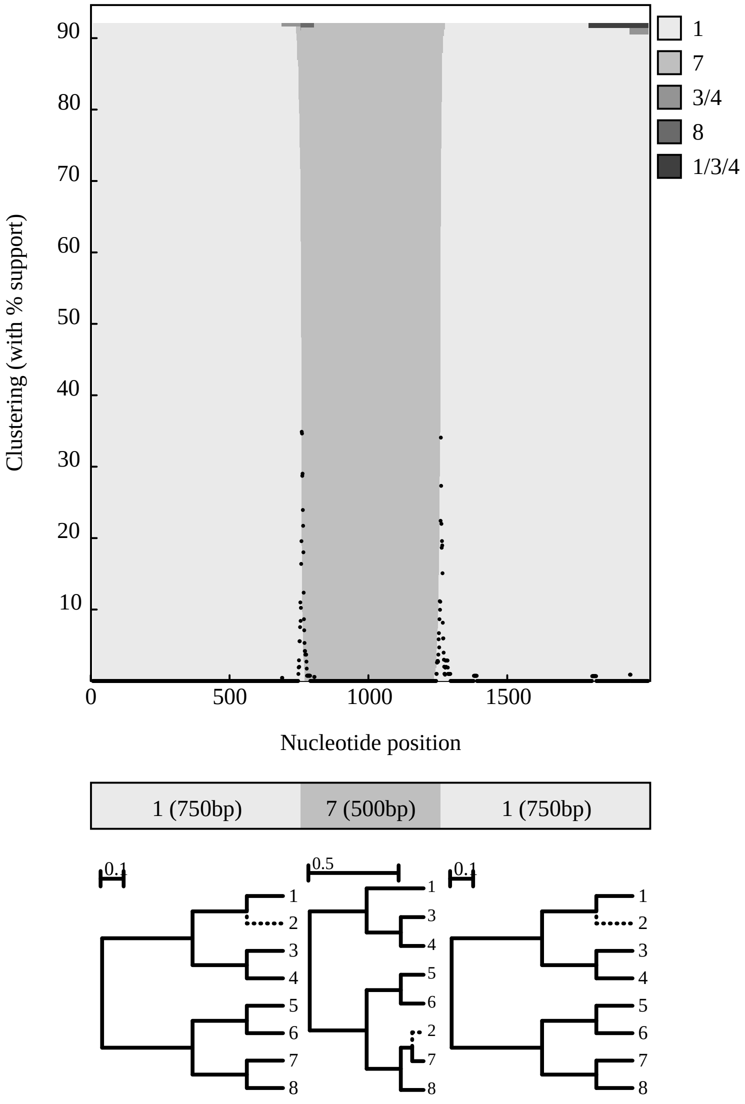


| Classification | Count | Better or Equal BIC/Delta(BIC) |
|---|---|---|
| Correct | 95 | 89/ 2.13 (-0.12,10.38) |
| Subset | 0 | |
| Superset | 5 | 5/ 6.75 ( 0.38,15.22) |
| Mismatch | 0 | |

| Breakpoint | Times found/ Location (median, st.dev (2.5%-97.5%)) |
|---|---|
| 750bp 1:7 | 98/751, 5.61 (735,762) |
| 1250bp 7:1 | 100/1251, 5.92 (1237,1265) |

Figure S6: Scenario 3. Two evident breakpoints/divergent

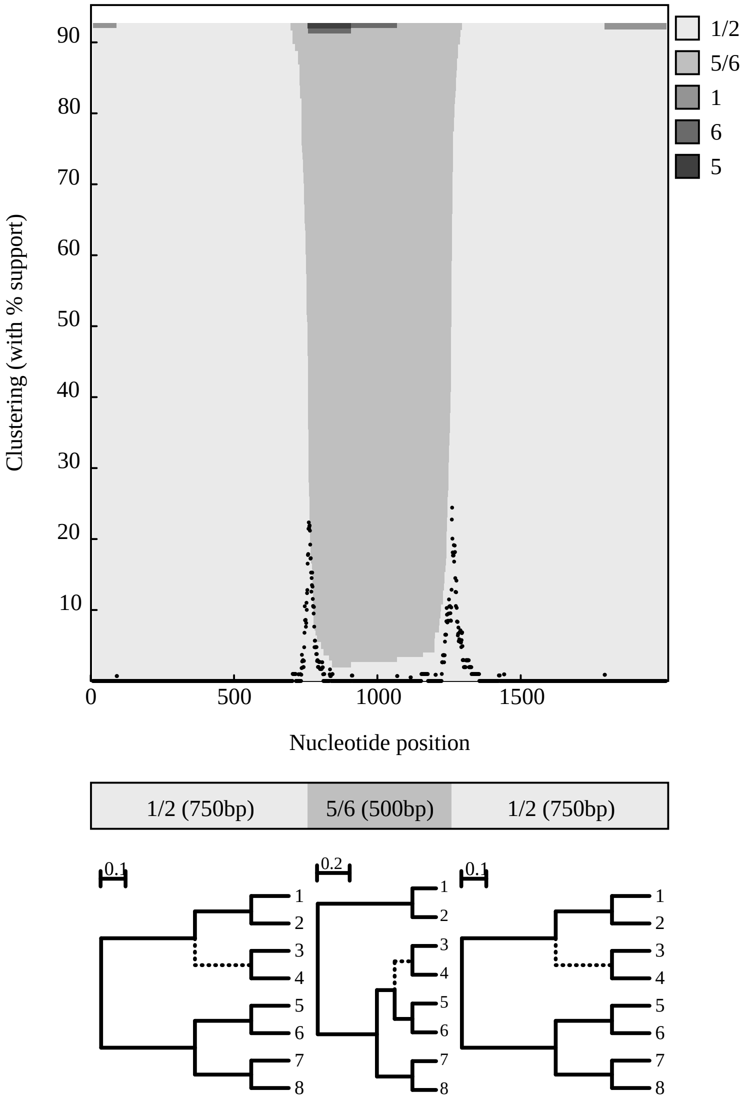


| Classification | Count | Better or Equal BIC/Delta(BIC) |
|---|---|---|
| Correct | 91 | 90/ 4.10 ( 0.31,14.70) |
| Subset | 0 | |
| Superset | 5 | 4/ 4.24 (-4.40,12.70) |
| Mismatch | 4 | 4/13.81 ( 0.67,15.30) |

| Breakpoint | Times found/ Location (median, st.dev (2.5%-97.5%)) |
|---|---|
| 750bp 1/2:5/6 | 96/749,22.37 (697,824) |
| 1250bp 5/6:1/2 | 96/1250,20.09 (1192,1283) |

Figure S7: Scenario 3. Two evident breakpoints/ancient

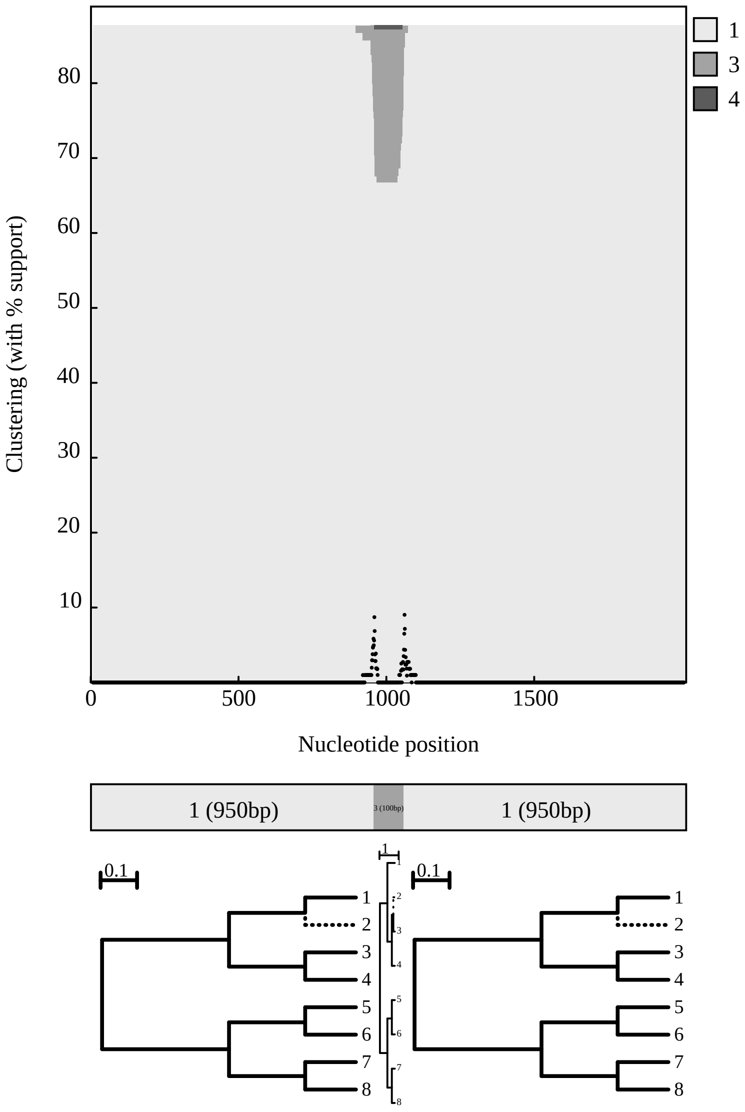


| Classification | Count | Better or Equal BIC/Delta(BIC) |
|---|---|---|
| Correct | 22 | 21/ 3.03 (-0.01,15.19) |
| Subset | 77 | 76/16.45 ( 2.02,53.45) |
| Superset | 0 | |
| Mismatch | 1 | 1/ 3.57 ( 3.57, 3.57) |

| Breakpoint | Times found/ Location (median, st.dev (2.5%-97.5%)) |
|---|---|
| 950bp 1:3 | 22/948,15.35 (888,960) |
| 1050bp 3:1 | 22/1050, 7.77 (1031,1066) |

Figure S8: Scenario 4. Two closely-spaced breakpoints/close

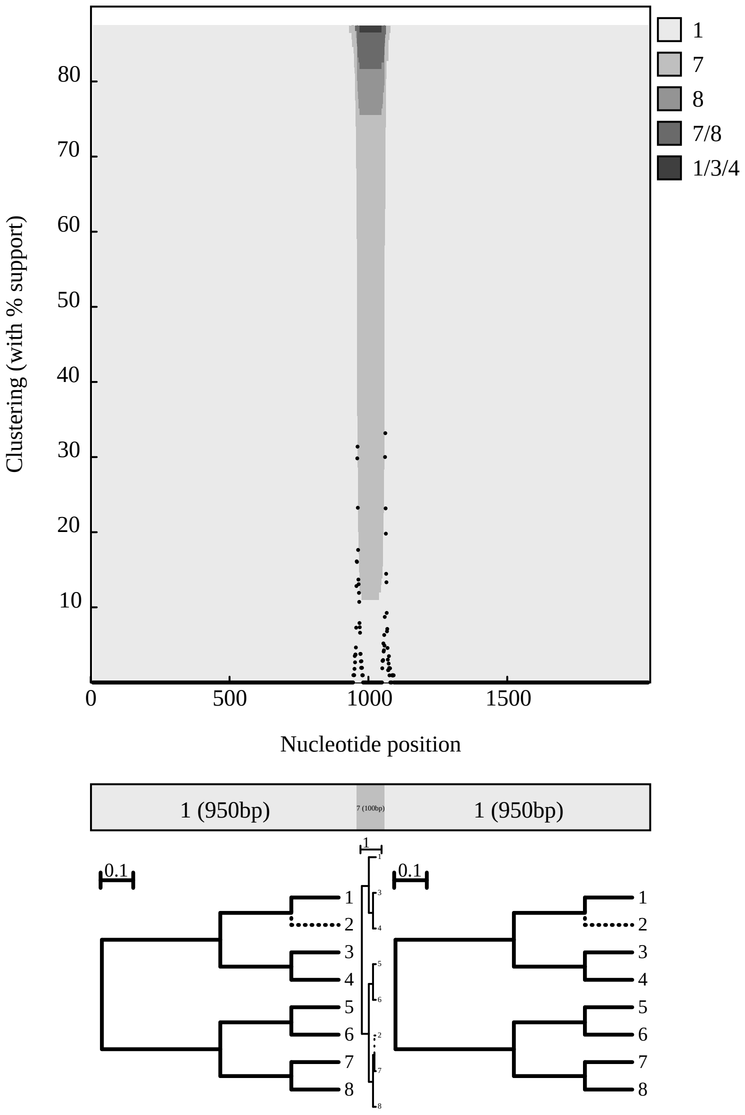


| Classification | Count | Better or Equal BIC/Delta(BIC) |
|---|---|---|
| Correct | 73 | 69/ 2.23 (-0.27,10.36) |
| Subset | 11 | 0/-33.93 (-71.70,-23.49) |
| Superset | 0 | |
| Mismatch | 16 | 15/ 4.37 (-0.28,14.95) |

| Breakpoint | Times found/ Location (median, st.dev (2.5%-97.5%)) |
|---|---|
| 950bp 1:7 | 73/951, 6.40 (932,960) |
| 1050bp 7:1 | 73/1051, 5.79 (1038,1068) |

Figure S9: Scenario 3. Two closely-spaced breakpoints/divergent

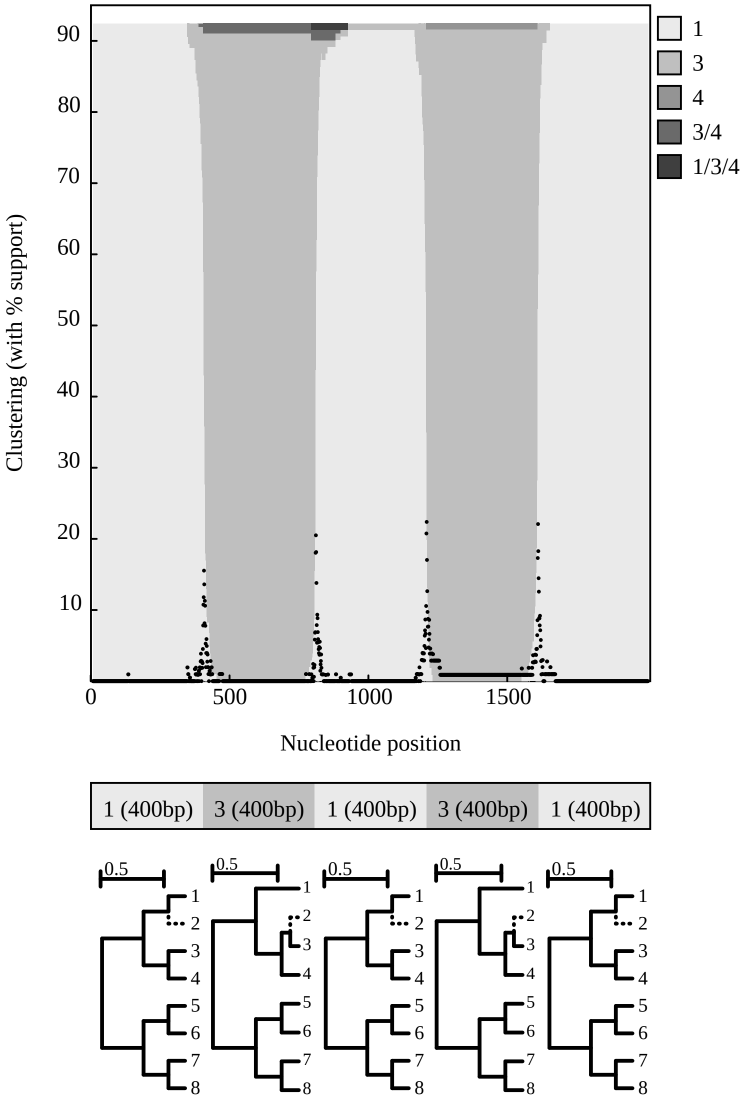


| Classification | Count | Better or Equal BIC/Delta(BIC) |
|---|---|---|
| Correct | 96 | 96/ 8.51 ( 0.36,22.19) |
| Subset | 0 | |
| Superset | 1 | 0/-15.08 (-15.08,-15.08) |
| Mismatch | 3 | 1/-0.53 (-12.70, 4.79) |

| Breakpoint | Times found/ Location (median, st.dev (2.5%-97.5%)) |
|---|---|
| 400bp 1:3 | 98/399,16.86 (342,428) |
| 800bp 3:1 | 97/803, 9.63 (784,837) |
| 1200bp 1:3 | 98/1200,11.34 (1161,1220) |
| 1600bp 3:1 | 99/1602,12.24 (1570,1634) |

Figure S10: Scenario 5. Four breakpoints/close

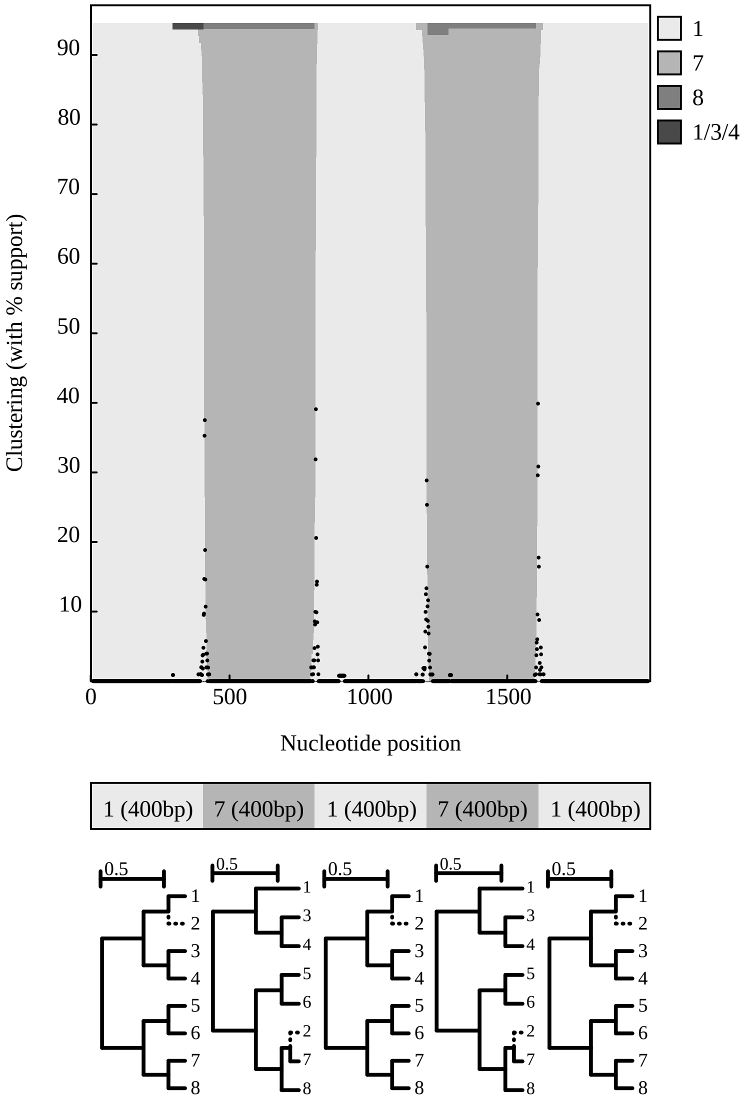


| Classification | Count | Better or Equal BIC/Delta(BIC) |
|---|---|---|
| Correct | 95 | 95/ 5.25 ( 0.23,20.04) |
| Subset | 0 | |
| Superset | 3 | 3/ 6.41 ( 2.06, 7.77) |
| Mismatch | 2 | 2/10.02 ( 5.48,14.57) |

| Breakpoint | Times found/ Location (median, st.dev (2.5%-97.5%)) |
|---|---|
| 400bp 1:7 | 98/401, 5.15 (389,412) |
| 800bp 7:1 | 99/802, 4.57 (787,809) |
| 1200bp 1:7 | 98/1201, 5.77 (1187,1211) |
| 1600bp 7:1 | 99/1602, 4.21 (1594,1613) |

Figure S11: Scenario 5. Four breakpoints/divergent

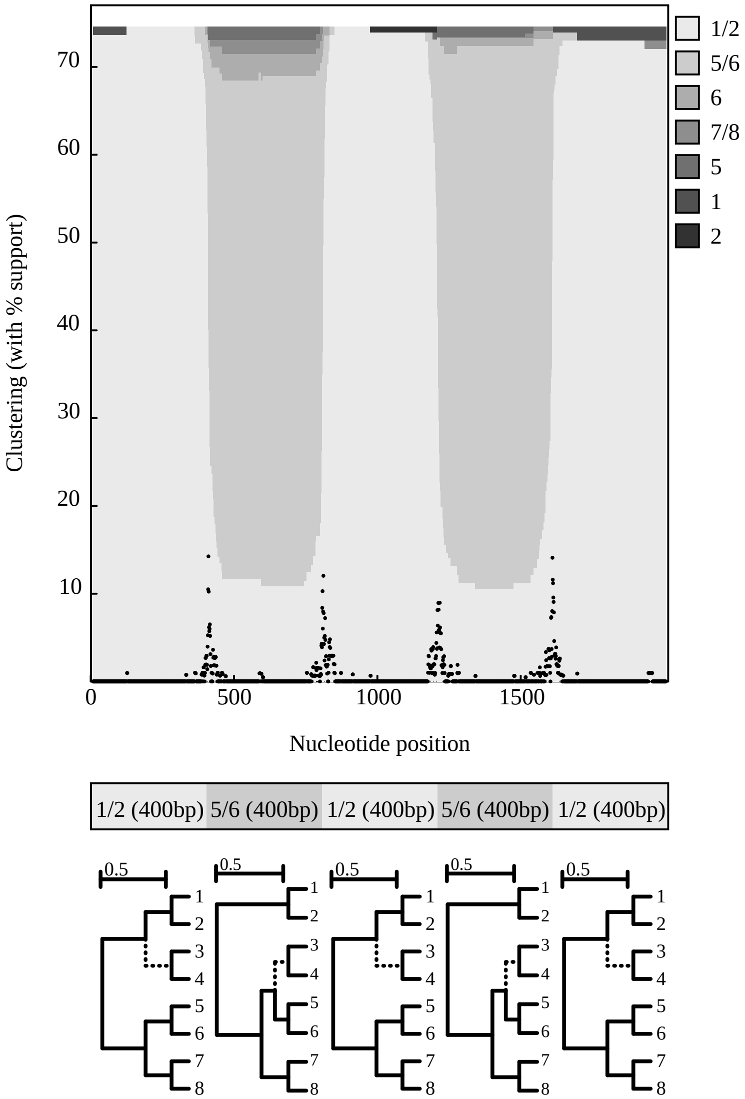


| Classification | Count | Better or Equal BIC/Delta(BIC) |
|---|---|---|
| Correct | 54 | 54/ 8.67 ( 0.68,27.76) |
| Subset | 22 | 3/-18.30 (-56.79,10.70) |
| Superset | 4 | 4/ 7.68 ( 5.06, 9.92) |
| Mismatch | 20 | 19/15.53 (-1.85,26.39) |

| Breakpoint | Times found/ Location (median, st.dev (2.5%-97.5%)) |
|---|---|
| 400bp 1/2:5/6 | 65/402,14.88 (357,434) |
| 800bp 5/6:1/2 | 67/802,15.74 (745,826) |
| 1200bp 1/2:5/6 | 69/1201,19.67 (1169,1270) |
| 1600bp 5/6:1/2 | 69/1602,18.04 (1550,1627) |

Figure S12: Scenario 5. Four breakpoints/ancient

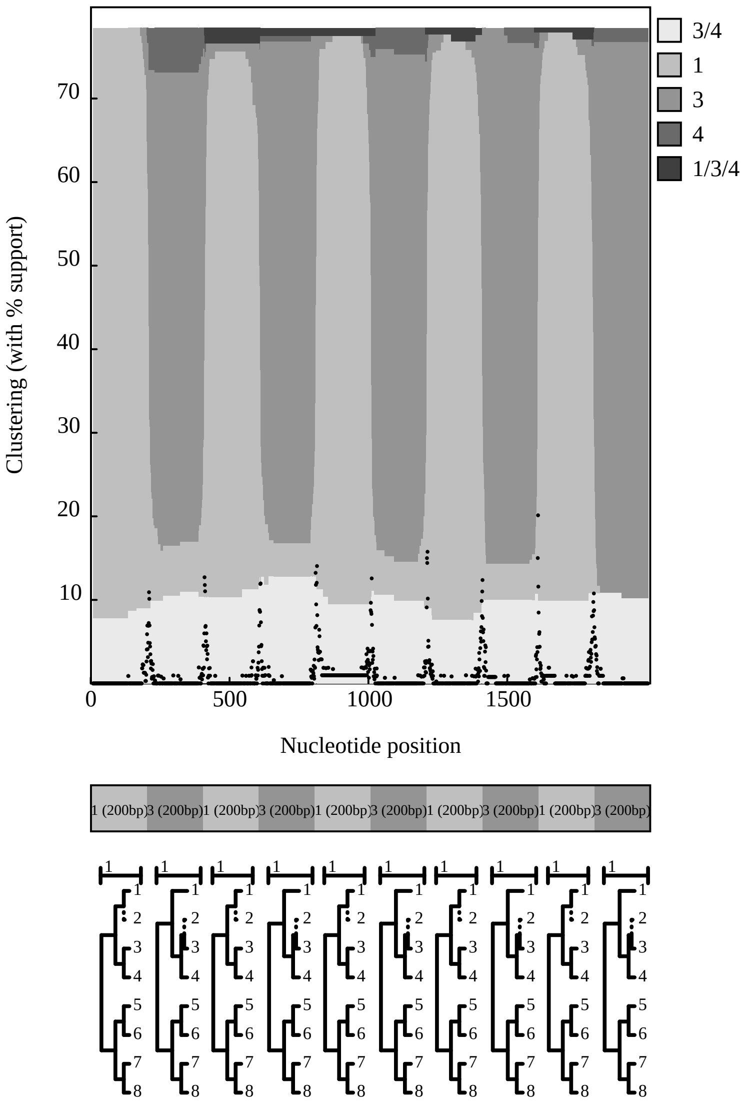


| Classification | Count | Better or Equal BIC/Delta(BIC) |
|---|---|---|
| Correct | 30 | 30/18.55 ( 6.99,38.23) |
| Subset | 13 | 2/-20.33 (-97.58,10.71) |
| Superset | 9 | 7/17.53 (-4.98,36.90) |
| Mismatch | 48 | 25/ 5.07 (-153.13,38.18) |

| Breakpoint | Times found/ Location (median, st.dev (2.5%-97.5%)) |
|---|---|
| 200bp 1:3 | 68/201,11.72 (176,235) |
| 400bp 3:1 | 62/403, 8.18 (391,420) |
| 600bp 1:3 | 68/601,14.69 (561,634) |
| 800bp 3:1 | 71/803,10.72 (783,838) |
| 1000bp 1:3 | 71/1001,10.95 (974,1021) |
| 1200bp 3:1 | 72/1203,13.14 (1177,1253) |
| 1400bp 1:3 | 75/1401,11.45 (1364,1414) |
| 1600bp 3:1 | 73/1602, 8.52 (1582,1626) |
| 1800bp 1:3 | 77/1801,13.40 (1746,1816) |

Figure S13: Scenario 6. Nine breakpoints/close

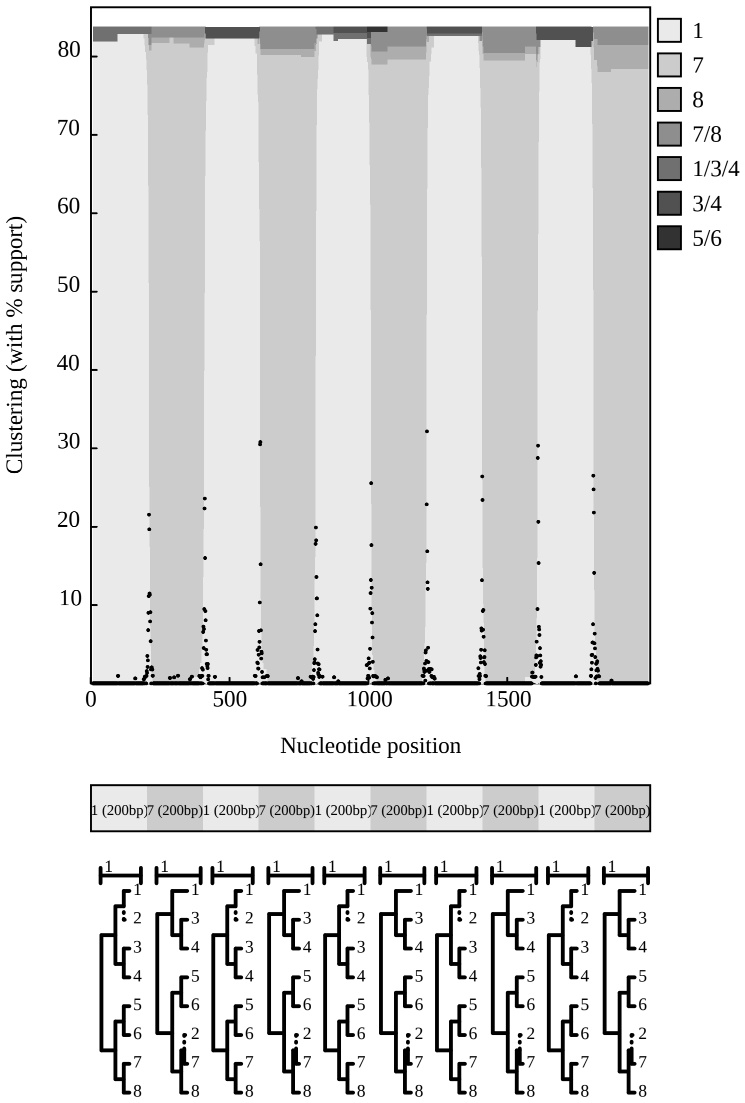


| Classification | Count | Better or Equal BIC/Delta(BIC) |
|---|---|---|
| Correct | 64 | 64/12.81 ( 1.78,40.35) |
| Subset | 0 | |
| Superset | 9 | 7/12.62 (-1.00,39.72) |
| Mismatch | 27 | 25/17.03 (-10.36,36.37) |

| Breakpoint | Times found/ Location (median, st.dev (2.5%-97.5%)) |
|---|---|
| 200bp 1:7 | 96/202, 4.87 (188,212) |
| 400bp 7:1 | 94/402, 7.98 (386,415) |
| 600bp 1:7 | 93/601, 5.95 (591,625) |
| 800bp 7:1 | 93/802, 5.37 (790,815) |
| 1000bp 1:7 | 92/1002, 5.35 (985,1015) |
| 1200bp 7:1 | 93/1202, 6.17 (1191,1228) |
| 1400bp 1:7 | 93/1402, 4.52 (1391,1411) |
| 1600bp 7:1 | 93/1602, 4.05 (1594,1612) |
| 1800bp 1:7 | 89/1802, 3.80 (1794,1814) |

Figure S14: Scenario 6. Nine breakpoints/divergent

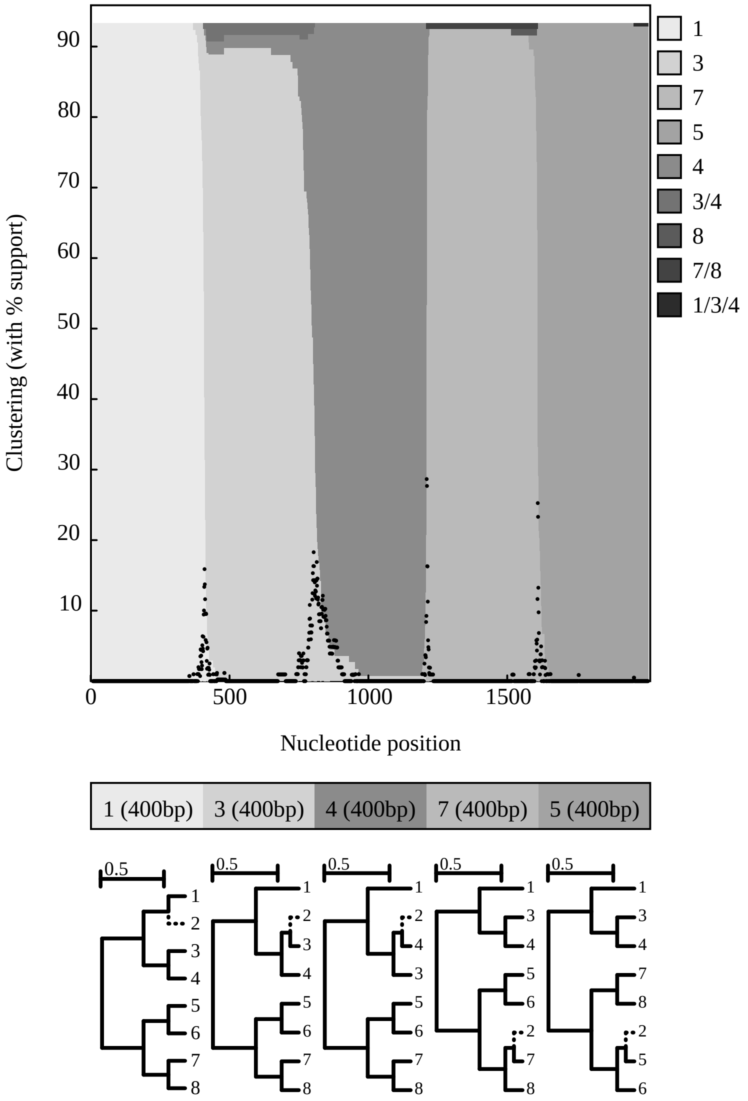


| Classification | Count | Better or Equal BIC/Delta(BIC) |
|---|---|---|
| Correct | 88 | 86/ 7.24 ( 0.36,21.66) |
| Subset | 3 | 1/-2.90 (-19.55, 2.77) |
| Superset | 5 | 4/ 5.69 (-0.79,10.58) |
| Mismatch | 4 | 4/ 3.85 ( 1.52,10.41) |

| Breakpoint | Times found/ Location (median, st.dev (2.5%-97.5%)) |
|---|---|
| 400bp 1:3 | 94/400,11.89 (375,440) |
| 800bp 3:4 | 89/793,28.29 (737,853) |
| 1200bp 4:7 | 98/1202, 4.02 (1192,1211) |
| 1600bp 7:5 | 98/1601.5,11.08 (1586,1640) |

Figure S15: Scenario 7. Complex mosaic

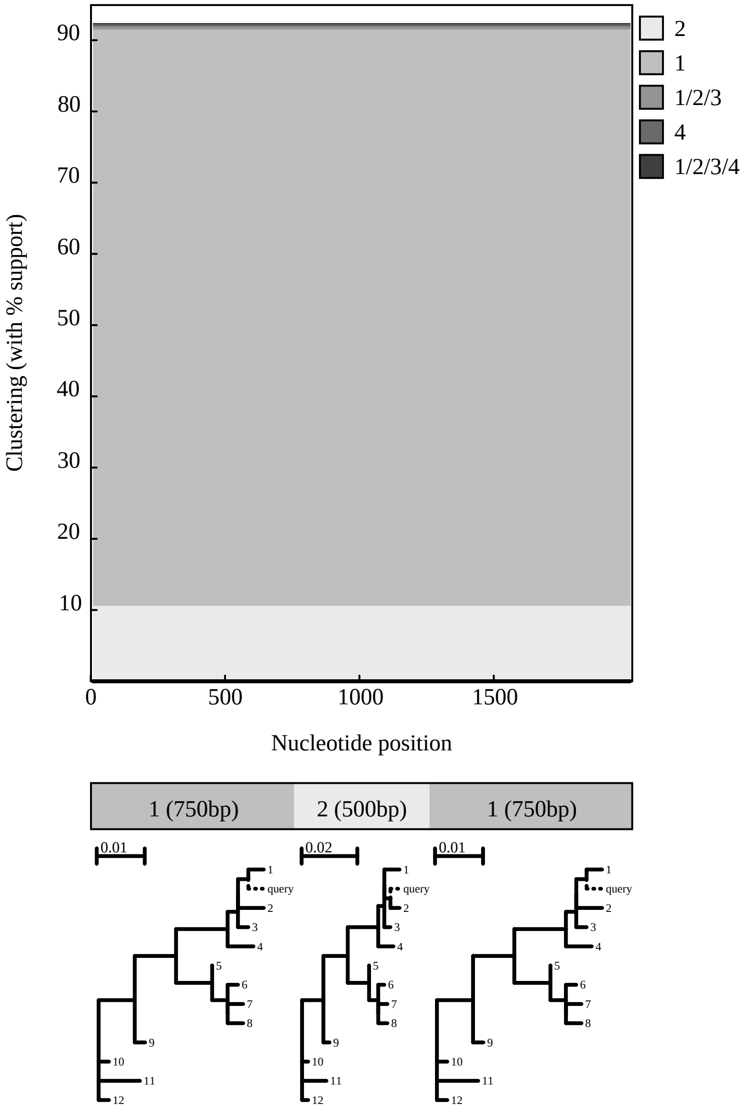


| Classification | Count | Better or Equal BIC/Delta(BIC) |
|---|---|---|
| Correct | 0 | |
| Subset | 96 | 96/59.53 (30.53,71.19) |
| Superset | 0 | |
| Mismatch | 4 | 4/70.57 (58.68,71.30) |

| Breakpoint | Times found/ Location (median, st.dev (2.5%-97.5%)) |
|---|---|
| 750bp 1:2 | |
| 1250bp 2:1 | |

Figure S16: Scenario 8. HIV within-patient 2 breakpoints /close

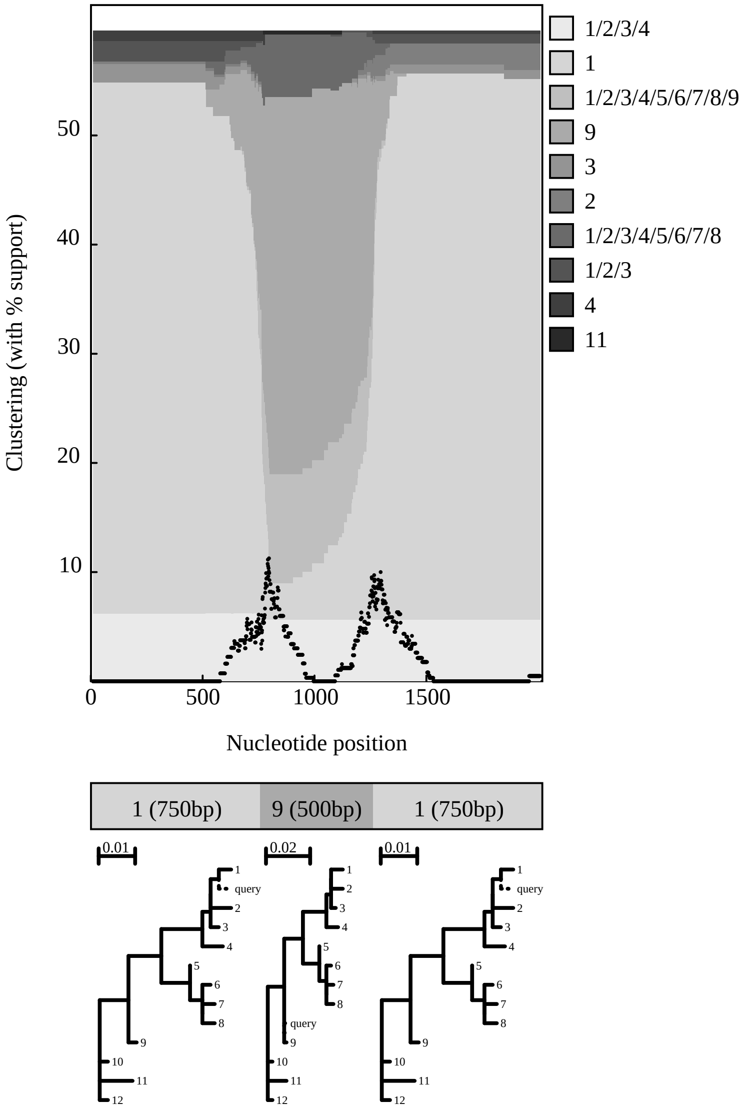


| Classification | Count | Better or Equal BIC/Delta(BIC) |
|---|---|---|
| Correct | 38 | 36/ 0.85 (-0.20, 9.06) |
| Subset | 4 | 2/-5.77 (-33.15,27.40) |
| Superset | 1 | 1/ 0.22 ( 0.22, 0.22) |
| Mismatch | 57 | 55/ 3.12 (-2.56,34.19) |

| Breakpoint | Times found/ Location (median, st.dev (2.5%-97.5%)) |
|---|---|
| 750bp 1:9 | 38/741.5,34.62 (666,790) |
| 1250bp 9:1 | 39/1256,36.11 (1156,1326) |

Figure S17: Scenario 8. HIV within-patient 2 breakpoints /divergent

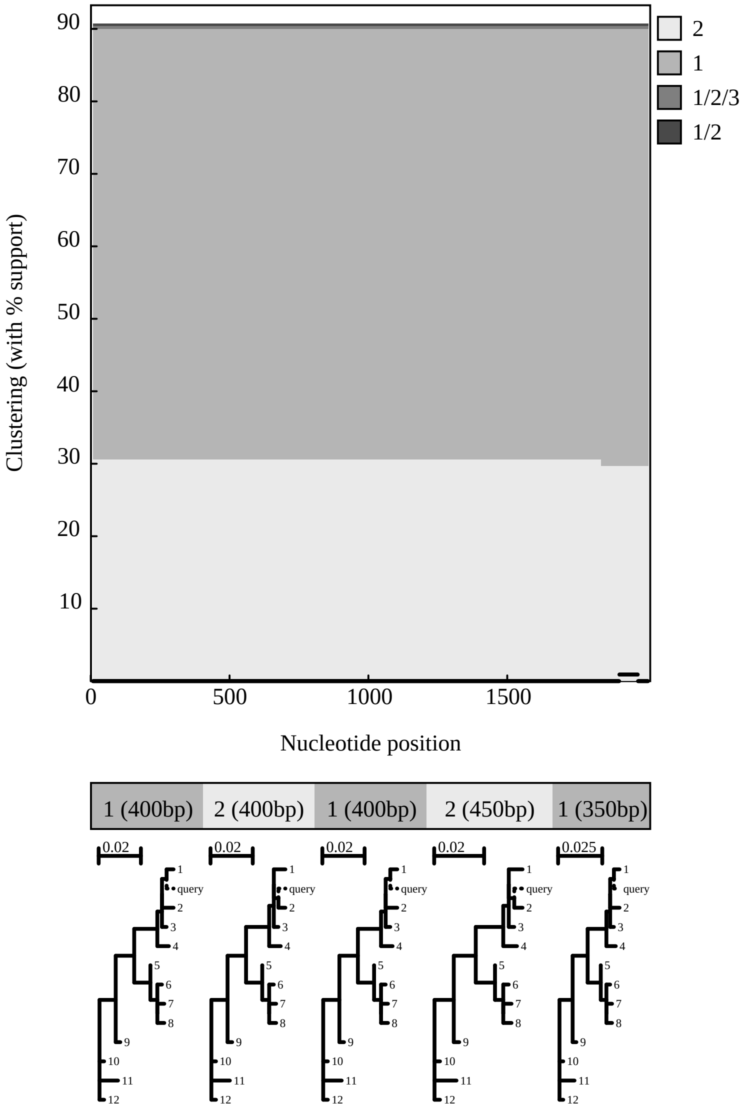


| Classification | Count | Better or Equal BIC/Delta(BIC) |
|---|---|---|
| Correct | 0 | |
| Subset | 97 | 97/97.20 (66.85,112.87) |
| Superset | 0 | |
| Mismatch | 3 | 3/110.97 (108.69,111.31) |

| Breakpoint | Times found/ Location (median, st.dev (2.5%-97.5%)) |
|---|---|
| 400bp 1:2 | |
| 800bp 2:1 | |
| 1200bp 1:2 | |
| 1650bp 2:1 | |

Figure S18: Scenario 9. HIV within-patient 4 breakpoints/close

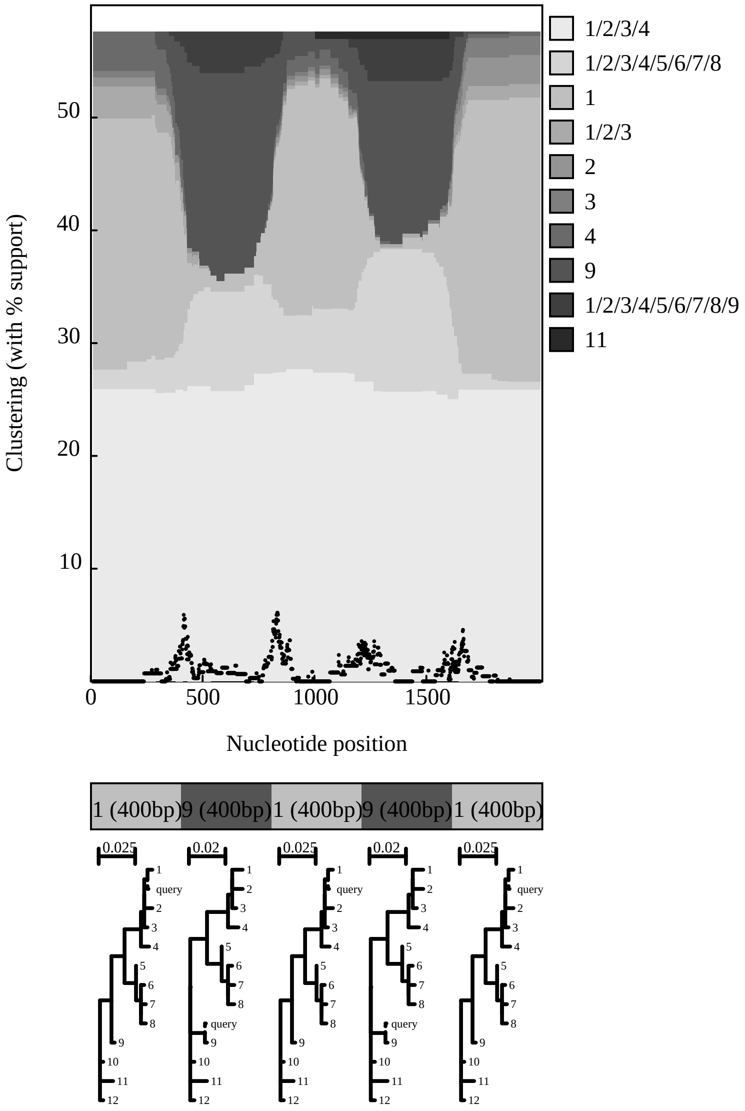


| Classification | Count | Better or Equal BIC/Delta(BIC) |
|---|---|---|
| Correct | 7 | 7/ 1.89 ( 1.00,11.18) |
| Subset | 2 | 1/ 8.45 (-12.30,29.19) |
| Superset | 1 | 0/-9.23 (-9.23,-9.23) |
| Mismatch | 90 | 70/ 5.64 (-43.44,46.21) |

| Breakpoint | Times found/ Location (median, st.dev (2.5%-97.5%)) |
|---|---|
| 400bp 1:9 | 16/391.5,32.00 (349,475) |
| 800bp 9:1 | 21/808,39.70 (730,868) |
| 1200bp 1:9 | 22/1202.5,42.90 (1118,1284) |
| 1600bp 9:1 | 20/1610.5,32.87 (1551,1676) |

Figure S19: Scenario 9. HIV within-patient 4 breakpoints/divergent

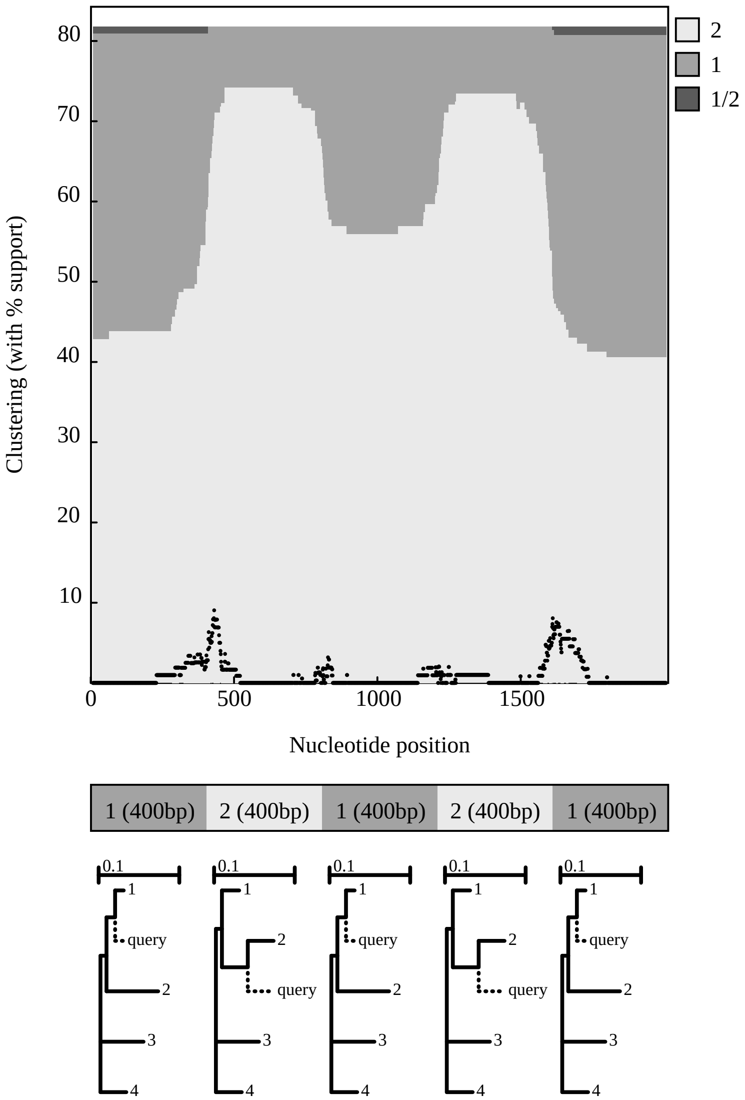


| Classification | Count | Better or Equal BIC/Delta(BIC) |
|---|---|---|
| Correct | 16 | 16/ 8.63 ( 0.52,27.53) |
| Subset | 80 | 77/22.80 (-1.94,54.31) |
| Superset | 0 | |
| Mismatch | 4 | 4/15.02 ( 8.96,28.68) |

| Breakpoint | Times found/ Location (median, st.dev (2.5%-97.5%)) |
|---|---|
| 400bp 1:2 | 30/402,31.53 (317,460) |
| 800bp 2:1 | 21/802,35.56 (716,885) |
| 1200bp 1:2 | 20/1209,30.18 (1151,1266) |
| 1600bp 2:1 | 35/1589,38.80 (1506,1689) |

Figure S20: Scenario 10. HIV intra-subtype

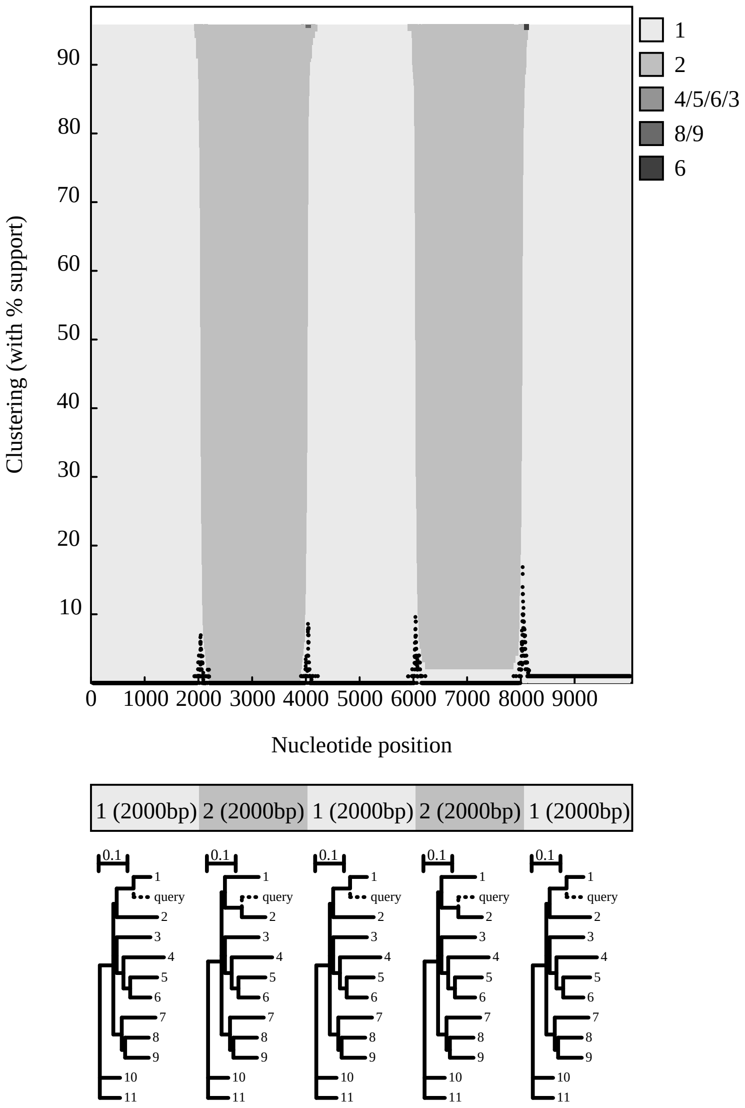


| Classification | Count | Better or Equal BIC/Delta(BIC) |
|---|---|---|
| Correct | 95 | 95/ 9.42 ( 0.38,23.26) |
| Subset | 1 | 0/-173.84 (-173.84,-173.84) |
| Superset | 2 | 2/17.40 (10.40,24.39) |
| Mismatch | 2 | 2/ 6.42 ( 5.98, 6.85) |

| Breakpoint | Times found/ Location (median, st.dev (2.5%-97.5%)) |
|---|---|
| 2000bp 1:2 | 94/2002.5,30.11 (1925,2092) |
| 4000bp 2:1 | 93/4000,29.62 (3928,4085) |
| 6000bp 1:2 | 94/6002.5,26.99 (5941,6067) |
| 8000bp 2:1 | 92/7996,33.39 (7929,8078) |

Figure S21: Scenario 11. HIV mosaic/close

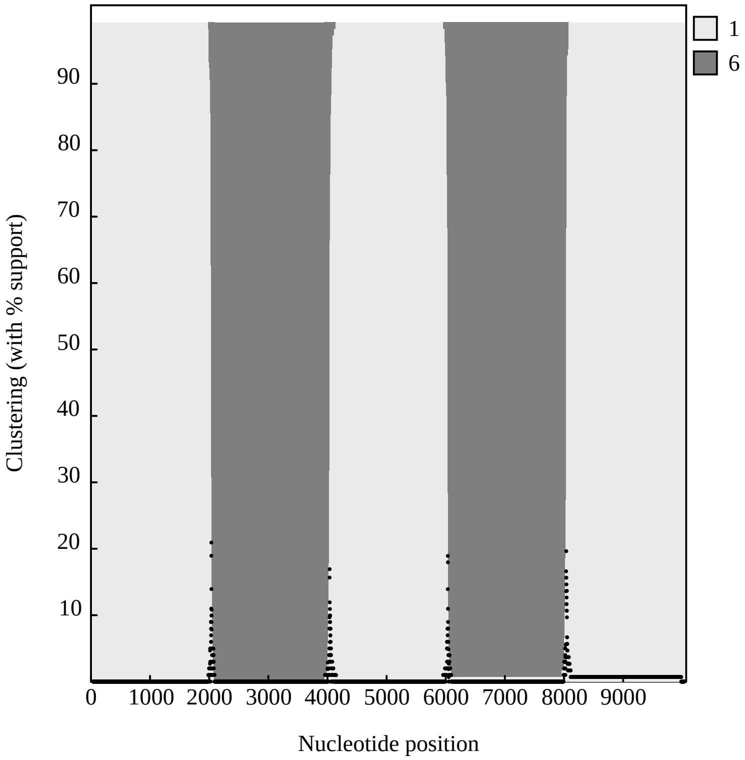


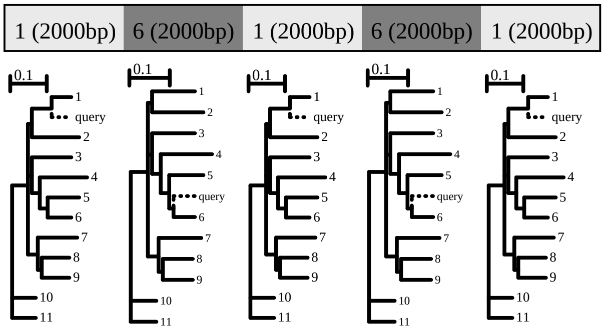


| Classification | Count | Better or Equal BIC/Delta(BIC) |
|---|---|---|
| Correct | 100 | 100/ 6.61 ( 0.34,19.94) |
| Subset | 0 | |
| Superset | 0 | |
| Mismatch | 0 | |

| Breakpoint | Times found/ Location (median, st.dev (2.5%-97.5%)) |
|---|---|
| 2000bp 1:6 | 100/2000,17.40 (1959,2042) |
| 4000bp 6:1 | 99/4003,21.62 (3964,4053) |
| 6000bp 1:6 | 100/6001,18.61 (5952,6040) |
| 8000bp 6:1 | 99/8004,16.88 (7968,8046) |

Figure S22: Scenario 11. HIV mosaic/medium

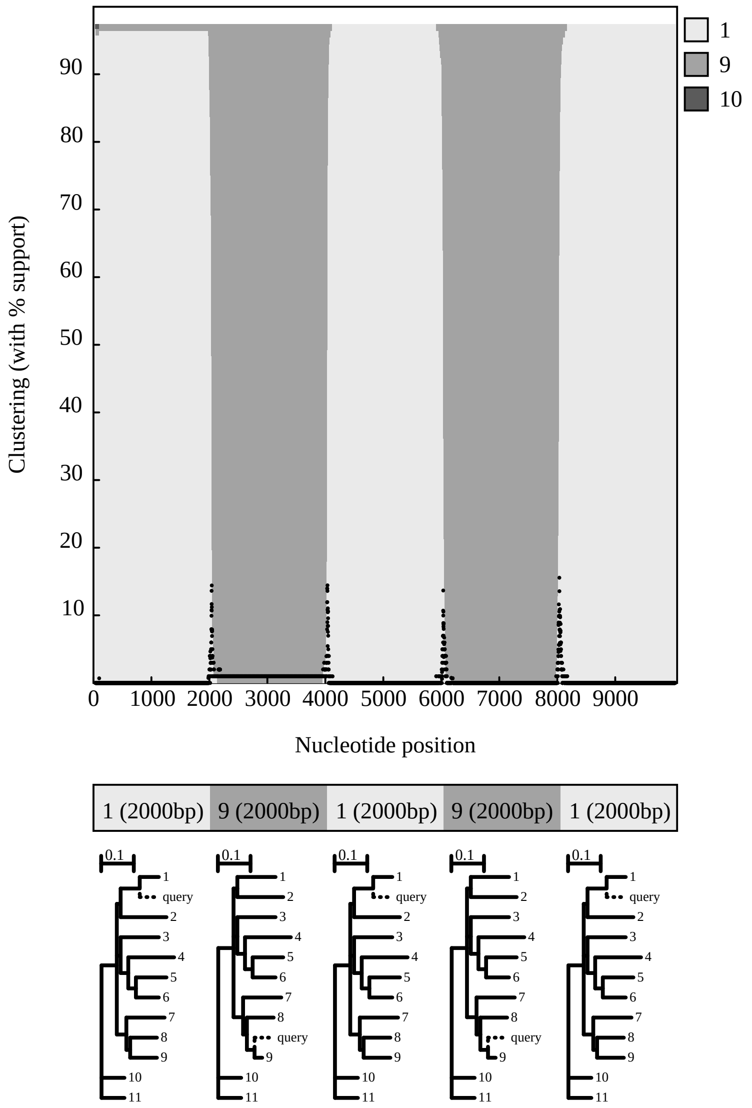


| Classification | Count | Better or Equal BIC/Delta(BIC) |
|---|---|---|
| Correct | 99 | 97/ 5.95 ( 0.12,19.81) |
| Subset | 0 | |
| Superset | 1 | 1/ 2.81 ( 2.81, 2.81) |
| Mismatch | 0 | |

| Breakpoint | Times found/ Location (median, st.dev (2.5%-97.5%)) |
|---|---|
| 2000bp 1:9 | 99/2002,20.92 (1956,2043) |
| 4000bp 9:1 | 100/4002.5,19.85 (3945,4056) |
| 6000bp 1:9 | 98/6000,21.89 (5937,6042) |
| 8000bp 9:1 | 99/7999,22.49 (7953,8070) |

Figure S23: Scenario 11. HIV mosaic/divergent

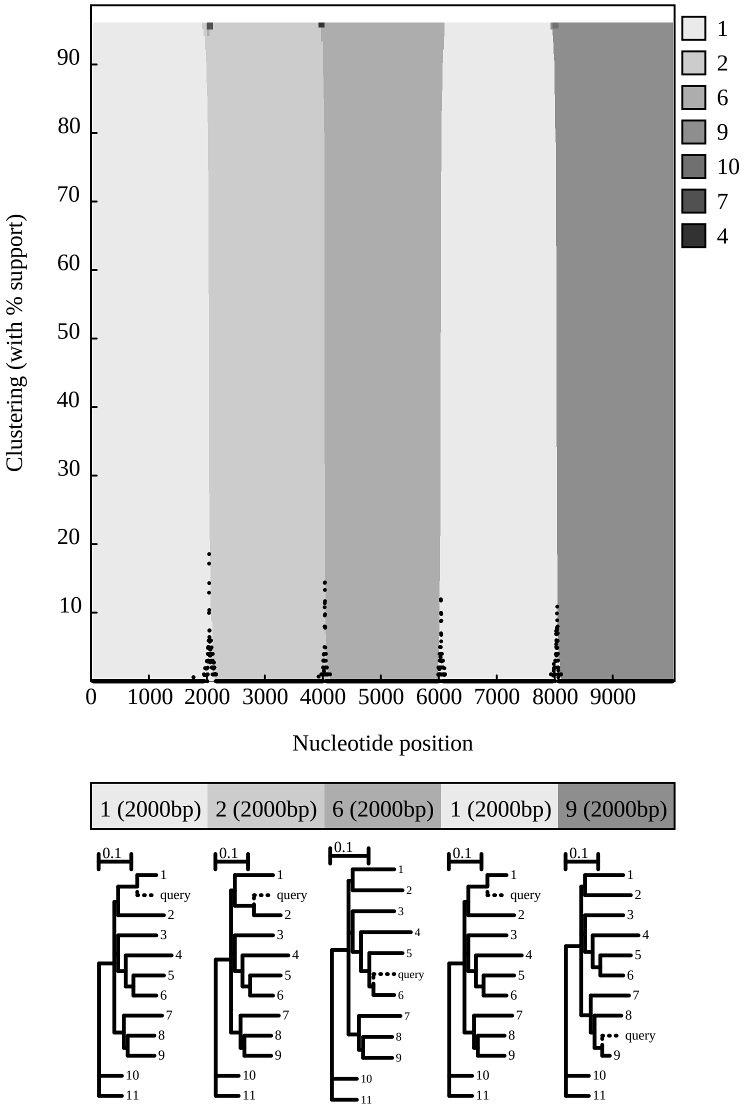


| Classification | Count | Better or Equal BIC/Delta(BIC) |
|---|---|---|
| Correct | 94 | 93/ 7.37 ( 0.59,21.03) |
| Subset | 0 | |
| Superset | 5 | 4/11.45 (-15.67,19.90) |
| Mismatch | 1 | 1/19.08 (19.08,19.08) |

| Breakpoint | Times found/ Location (median, st.dev (2.5%-97.5%)) |
|---|---|
| 2000bp 1:2 | 96/2003,27.61 (1940,2070) |
| 4000bp 2:6 | 99/4000,18.14 (3969,4053) |
| 6000bp 6:1 | 100/6003,20.35 (5959,6068) |
| 8000bp 1:9 | 97/8000,21.34 (7947,8062) |

Figure S24: Scenario 11. HIV mosaic/complex
